# Uncovering the Most Kinetically Influential Reaction Pathway Driving the Generation of HCN from Oxyma/DIC Adduct: A Theoretical Study

**DOI:** 10.1021/acs.iecr.2c03145

**Published:** 2023-01-05

**Authors:** Lingfeng Gui, Claire S. Adjiman, Amparo Galindo, Fareed Bhasha Sayyed, Stanley P. Kolis, Alan Armstrong

**Affiliations:** † Department of Chemical Engineering, The Sargent Centre for Process Systems Engineering and Institute for Molecular Science and Engineering, Imperial College London, LondonSW7 2AZ, U.K.; ‡ Synthetic Molecule Design and Development, Eli Lilly Services India Pvt Ltd, Devarabeesanahalli, Bengaluru560103, India; ¶ Synthetic Molecule Design and Development, Eli Lilly and Company, Lilly Corporate Center, Indianapolis, Indiana46285, United States; § Department of Chemistry and Institute for Molecular Science and Engineering, Imperial College London, Molecular Sciences Research Hub, White City Campus, LondonW12 0BZ, U.K.

## Abstract

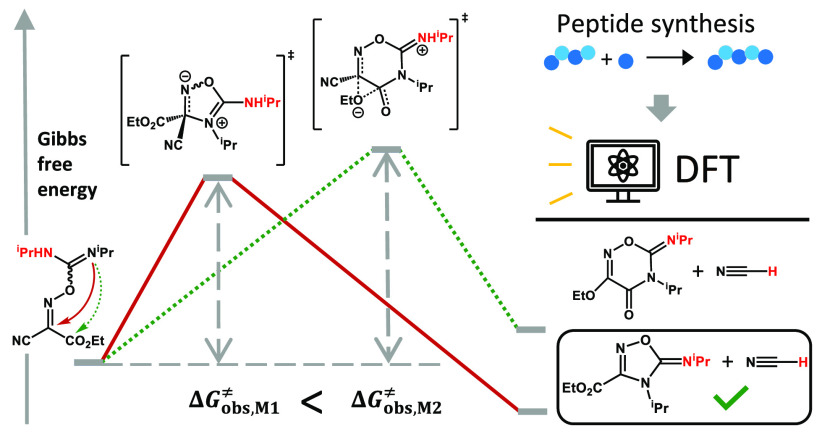

The combination of ethyl (hydroxyimino)cyanoacetate (Oxyma) and diisopropylcarbodiimide (DIC) has demonstrated superior performance in amino acid activation for peptide synthesis. However, it was recently reported that Oxyma and DIC could react to generate undesired hydrogen cyanide (HCN) at 20 °C, raising safety concerns for the practical use of this activation strategy. To help minimize the risks, there is a need for a comprehensive investigation of the mechanism and kinetics of the generation of HCN. Here we show the results of the first systematic computational study of the underpinning mechanism, including comparisons with experimental data. Two pathways for the decomposition of the Oxyma/DIC adduct are derived to account for the generation of HCN and its accompanying cyclic product. These two mechanisms differ in the electrophilic carbon atom attacked by the nucleophilic sp^2^-nitrogen in the cyclization step and in the cyclic product generated. On the basis of computed “observed” activation energies, Δ*G*_obs_^⧧^, the mechanism that proceeds via the attack of the sp^2^-nitrogen at the oxime carbon is identified as the most kinetically favorable one, a conclusion that is supported by closer agreement between predicted and experimental ^13^C NMR data. These results can provide a theoretical basis to develop a design strategy for suppressing HCN generation when using Oxyma/DIC for amino acid activation.

## Introduction

**Scheme 1:**
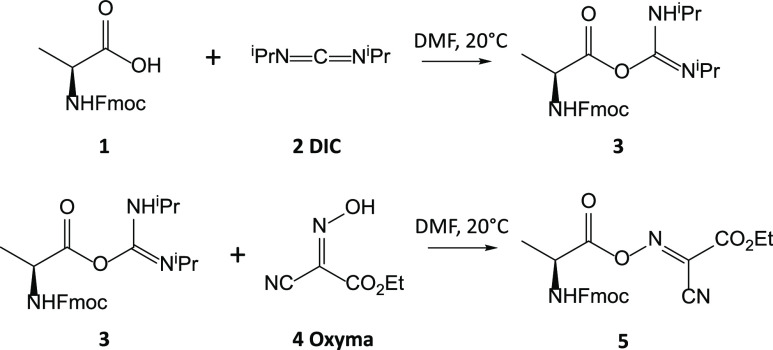
Reactions of the Oxyma (**4**)/DIC (**2**) Reagent Combination with an Amino Acid (**1**) to Form an Active Oxime Ester (**5**)

**Scheme 2:**
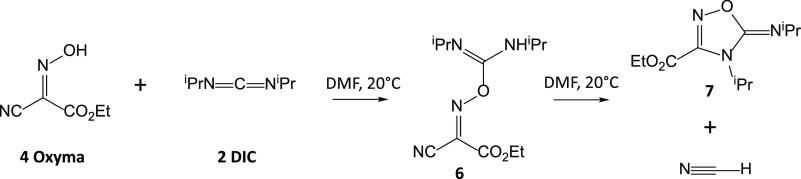
Reaction of Oxyma (**4**) and DIC (**2**) to Form a Linear Oxyma/DIC adduct (**6**) and Its Decomposition into HCN and a Five-Membered Ring Product (**7**)

Carboxylic acid activation is an important step in forming the amide linkage between two amino acids, and thus has wide applications in the synthesis of peptides and other polymers.^1^ In carboxylic acid activation (Scheme 1), amino acid **1** reacts with diisopropylcarbodiimide (DIC) **2** to form a strongly activated O-acylisourea intermediate **3** which then reacts with ethyl (hydroxyimino)cyanoacetate (Oxyma) **4** to form an active oxime ester **5**. Oxime ester **5** further reacts with the amino group to form a peptide bond such that the amino acid’s chirality is largely retained.^2−4^ The Oxyma/DIC reagent combination has been demonstrated to be a superior reagent combination for amino acid activation with the merits of high coupling efficiency, inhibition of racemization, and lower risk of explosion.^5^ However, it has recently been reported^6^ that Oxyma and DIC can undergo an intermolecular reaction, and generate hydrogen cyanide (HCN) during the process of amino acid activation in DMF at 20 °C. This can pose a serious threat to any personnel carrying out this reaction. It has been suggested that Oxyma and DIC first undergo an intermolecular reaction in an analogous manner to amino acid activation by DIC to form an acyclic linear adduct **6** that can quickly decompose into a cyclic structure **7** and HCN (Scheme 2). The five-membered ring structure **7** has been assigned based on NMR spectroscopy and LC-HRMS.^6^ Despite the instability of the intermediate **6** at room temperature, it has been detected with in situ NMR when the temperature is lowered to −30 °C. The decomposition reaction mechanism has been postulated by McFarland et al.^6^ to be an intramolecular nucleophilic attack on the oxime carbon by the sp^3^-nitrogen. However, this mechanism has not been verified experimentally or computationally.

Erny et al.^7^ attempted to reduce the risk of HCN generation from the reaction of Oxyma and DIC by selecting a different reaction solvent and scavenging produced HCN with dimethyl trisulfide. However, they found that HCN formation cannot be fully suppressed in this way with continued and significant safety concerns when the reaction is scaled up. They investigated the possible involvement of an N-oxyl radical from Oxyma in the reaction mechanism, but this possibility was excluded since the addition of diisopropylthiourea (DITU) as an N-oxyl radical scavenger did not affect the production of HCN. Manne et al.^8^ found that the steric hindrance caused by the side chains bonded to the nitrogen atoms of the carbodiimide has a large effect on HCN formation: the carbodiimide with two tertiary alkyl substituents, i.e., *N*,*N*′-di-*tert*-butylcarbodiimide (DTBC), does not lead to HCN formation but shows unacceptably poor performance in peptide synthesis. The carbodiimide with two primary alkyl substituents, i.e., *N*-ethyl-*N*′-(3-(dimethylamino)propyl)carbodiimide hydrochloride (EDC.HCl), forms no HCN with Oxyma but using it in peptide synthesis is still accompanied by a reduction in the purity of the peptide synthesized compared to DIC. The carbodiimide with the combination of one primary alkyl substituent and one tertiary substituent, i.e., *tert*-butylethylcarbodiimide (TBEC), can achieve similar or even better performance than DIC does, though currently TBEC is more expensive and its properties need to be further investigated before TBEC can replace DIC in the industrial manufacture of peptides.^9^ In another study, Manne et al.^10^ proposed a safer experimental protocol that can avoid the production of HCN. The amino acid is preactivated with DIC for 5 min, and the mixture is then added to peptide resin, 15 s after which Oxyma is added. However, this protocol is also likely to increase the chance of racemization during the process of preactivation.

To address the safety issues caused by HCN generation without compromising the performance of peptide synthesis, deeper insights into the reaction mechanism and kinetics are necessary. The objective of the current work is to investigate systematically mechanistic and kinetic aspects of the addition reaction of Oxyma and DIC and of the decomposition reaction of the Oxyma/DIC adduct **6** using density functional theory (DFT) calculations. Furthermore, a theoretical analysis is performed to identify the rate-determining step (RDS) that best accounts for the kinetics of the HCN generation.

## Computational Methods

All calculations are performed using B3LYP-D3^11^/6-31+g(d) in Gaussian 16, Revision C.01^12^. Here Grimme’s D3 dispersion^11^ is used to model the London dispersion interactions between species to ensure chemical accuracy. The default “ULTRAFINE” integral grid is used. The keyword “VTIGHT” is specified. Frequency calculations are performed at a temperature of 293 K to compute the thermal contributions to the Gibbs free energy and confirm that there is no imaginary frequency for the structures of reactants, products, and intermediates, and only one imaginary frequency for the structures of transition states. The transition state structures are further confirmed by running Intrinsic Reaction Coordinate (IRC)^13^ calculations to check whether the transition-state structures connect the corresponding reactants and products. The SMD continuum solvation model^14^ is utilized to simulate the solvent environment (*N*,*N*-dimethylformamide) implicitly in geometry optimization as well as NMR calculations. NMR spectra are computed using the Gauge-Independent Atomic Orbital (GIAO) method^15^ by specifying the keyword “NMR” in the Gaussian input files. A conformer search of **6** and **7** is conducted using the GMMX add-on in Gaussview 6 with the force field MMFF94.^16^ The resulting structures are then optimized with the DFT method at the aforementioned level of theory. Throughout the paper, Gibbs free energies are considered for the discussion.

## Results and Discussion

### The Addition Reaction of Oxyma and DIC

**Scheme 3:**
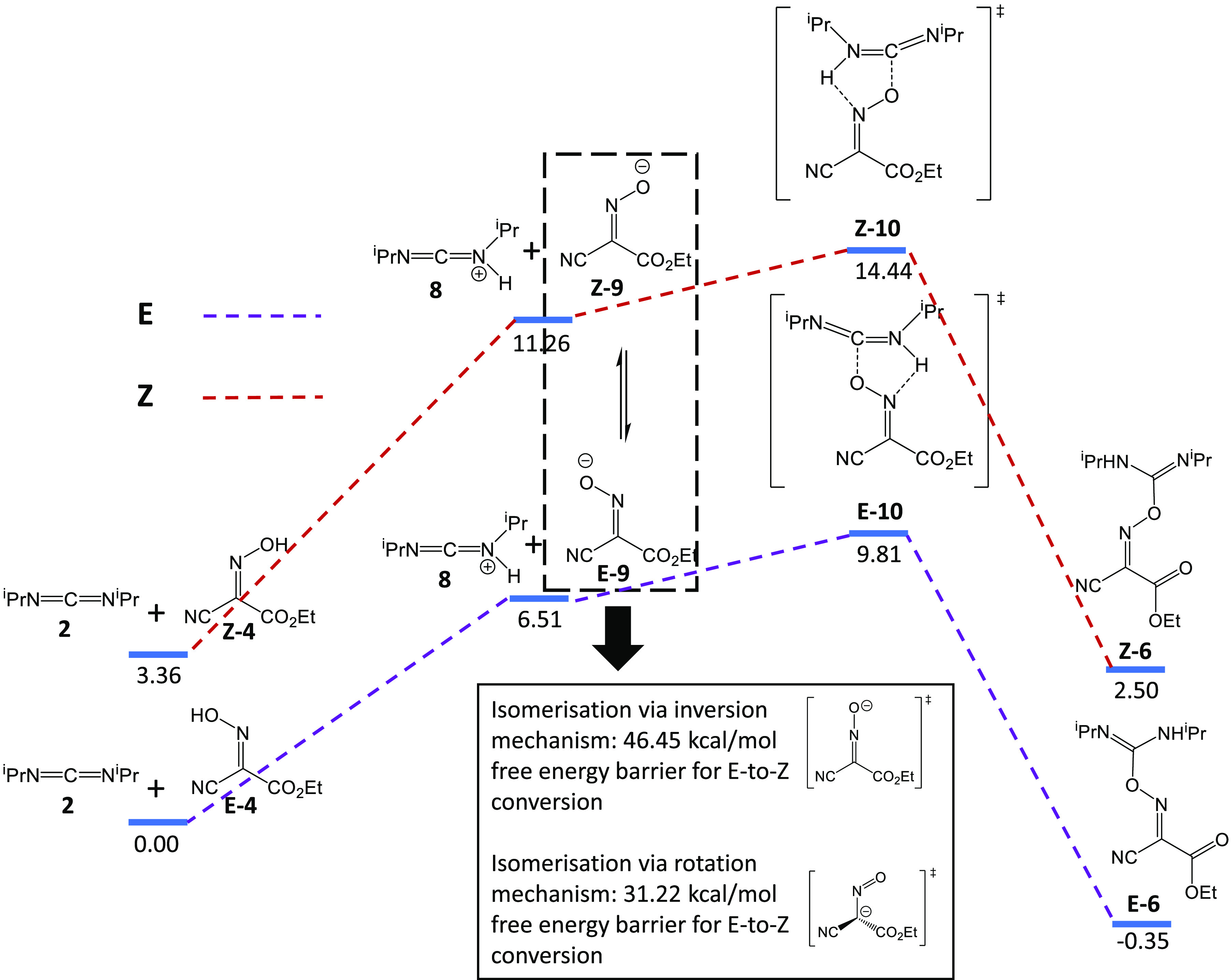
Schematic Representation of Mechanism of the Addition Reaction of Oxyma and DIC The black boxes (dashed and solid) correspond to the isomerization reaction between Z-**9** and E-**9** (The number under each blue bar in the scheme is the Gibbs free energy of the corresponding species in the unit of kcal/mol.).

We first investigate the mechanism of the addition reaction of Oxyma and DIC, as it is considered to be similar to that of an amino acid and DIC.^6^ It has previously been proposed that the reaction of an amino acid and DIC begins with a proton transfer from the carboxylic acid group of an amino acid to one of the DIC nitrogens, followed by the nucleophic attack of the deprotonated amino acid anion on the central carbon of the protonated DIC.^17^ A similar reaction process is modeled here carrying out DFT calculations, with the amino acid replaced by Oxyma as shown in Scheme 3. Since Oxyma has two oxime bond configurations (E- and Z-), and the energetics of the reactions starting from these two configurations differ, the calculations are performed for each configuration. In both cases, as seen in Scheme 3, the Gibbs free energy of the transition states Z/E-**10** of the nucleophilic-addition step relative to either the neutral reactants **2** and E/Z-**4** or their ion pair **8** and Z/E-**9** are found to be small enough for the reaction to take place at room temperature, with the free energy barrier for the E-oxime configuration being slightly lower than that for the Z-oxime configuration. The free energy barrier here refers to the free energy difference between Z-/E-**10** and the neutral pair of Oxyma and DIC. The potential energy surface is scanned for the proton exchange reaction, but the potential energy keeps rising without a saddle point detected when the proton moves away from Oxyma toward DIC.

It is useful to assess whether the oxime bond can interconvert between its E- and Z-configurations since this can help determine the ratio of E- and Z-oxime adduct **6** generated by the addition reaction. It has been reported that oxime bonds do not usually undergo isomerization at room temperature. The oxime bond has great configurational stability under thermal conditions; for example, the *o*-methyl ether of cis-*p*-chlorobenzophenone oxime has been found not to isomerize after being heated for 170 h at 230 °C.^18,19^ However, isomerization may be easier for the anionic intermediate **9** due to delocalization (resonance) that might reduce the bond order of the C=N double bond. To investigate this, we locate two transition states for the isomerization of E- and Z-**9** (Scheme 3). One transition state, corresponding to the rotation mechanism, requires an activation energy of 31.2 kcal/mol, which is inaccessible at room temperature while the other, corresponding to an inversion at the nitrogen atom requires an even higher activation energy of 46.5 kcal/mol. From these energetics, it can be concluded that it is unlikely for either E-**9** or Z-**9** to isomerize at room temperature. Consequently, the oxime bond configuration of adduct **6** will depend on the initial configuration of Oxyma which is unknown, although it has been suggested that Oxyma is predominantly in its Z-oxime configuration.^20^

Similar to the addition of the ion pair **8** and Z/E-**9**, the Gibbs free energy barrier for the reverse reaction is also low, which indicates that the reaction is reversible with respect to the subsequent cyclization reaction. Thus, it is important to understand the cyclization pathway so that the RDS can be determined in order to minimize the amount of HCN generated. In the following section, we focus on the mechanism of the decomposition reaction of the Oxyma/DIC adduct **6**.

### The Decomposition Reaction of the Oxyma/DIC Adduct **6**

**Figure 1:**
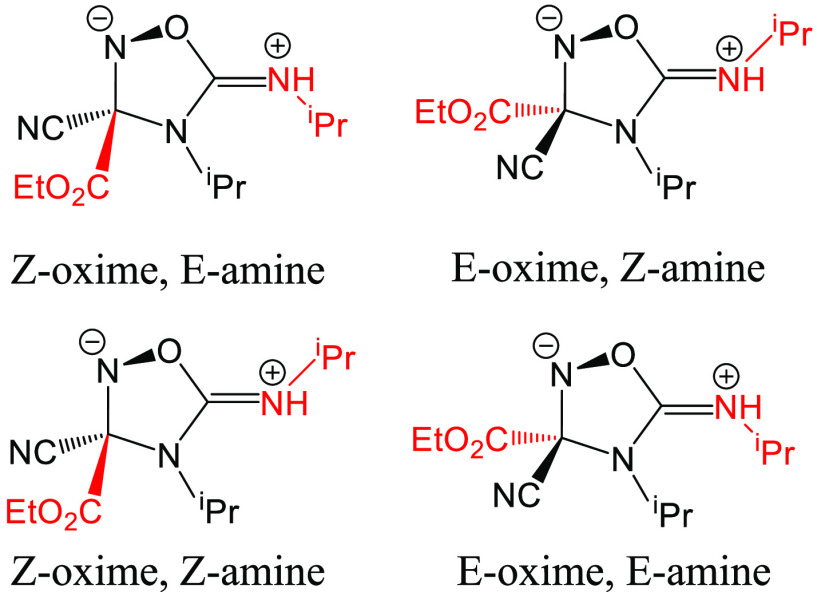
Representation of four configurations of the cyclic intermediate formed by the initial cyclization of Oxyma/DIC adduct **6**. The Z-oxime configuration indicates that the N–O bond and the C–CN bond are on different sides of the ring plane, as is expected to result from the cyclization of a Z-oxime **6**, while the E-oxime configuration indicates both bonds are on the same side of the plane; the denoted amine configuration refers to the configuration of the C=NH^i^Pr bond).

To form the five-membered ring product **7**, the initial cyclization step requires one of the nitrogens originating from DIC to attack the oxime carbon. The sp^2^-nitrogen is likely to be more nucleophilic toward cyclization because the resulting charge can be stabilized by resonance. Therefore, in the following analysis, we focus only on the nucleophilic attack by the sp^2^-nitrogen (Mechanism **M1**). We have also studied the mechanisms involving nucleophilic attack by the sp^3^-nitrogen, Mechanism **M3** and Mechanism **M4**, which are found to have considerably higher energy barriers, and they are discussed in the Supporting Information. It should be noted that the reaction system is highly flexible, which results in many similar reaction pathways that differ on the basis of conformation. Among them, the minimum energy reaction path (MERP) is the most representative one as (exponentially) more species are likely to follow this path. To identify the MERP, the cyclic intermediate (Figure 1) formed in the initial cyclization is used to characterize each reaction pathway since the corresponding transition state of the cyclization step, and further, the whole reaction pathway, can be derived by specifying the relevant stereoisomer of the cyclic intermediate. To obtain all the stereoisomers of this cyclic intermediate, a relaxed multidimensional potential energy surface scan is carried out for each of the configurations shown in Figure 1. The details of the derivation and the search can be found in the Supporting Information.

### Mechanism **M1**: Nucleophilic Attack by the sp^2^-Nitrogen at the Oxime Carbon

**Scheme 4:**
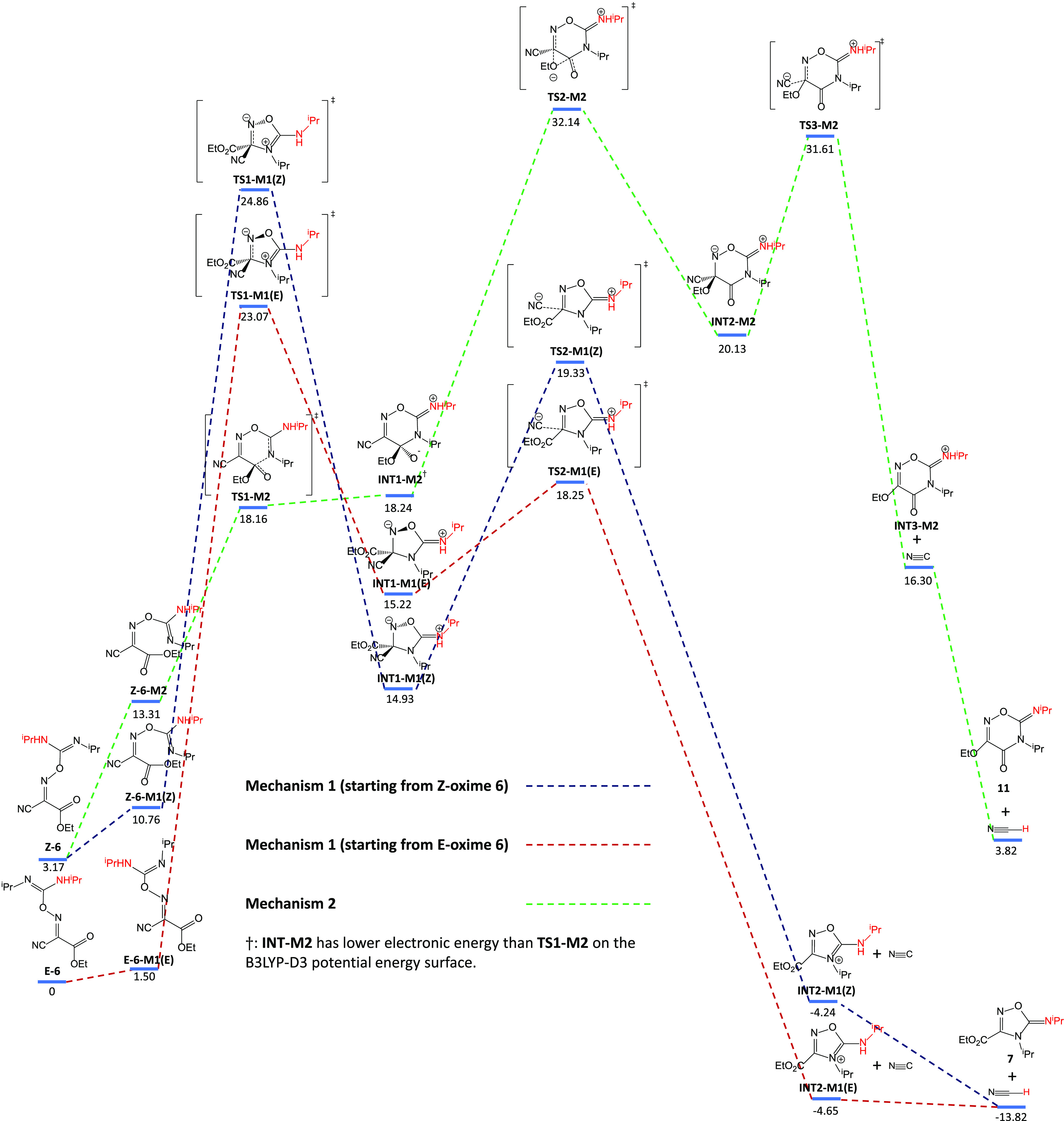
Schematic Representation of Mechanism **M1** Starting from **Z-6** and **E-6**, and Mechanism **M2** The number under each blue bar in the scheme is the Gibbs free energy of the corresponding species in the unit of kcal/mol.

In Scheme 4 we show **M1**(E) and **M1**(Z), the MERPs for the cyclization reaction from E-**6** and Z-**6**, respectively. In **M1**, the C=N^i^Pr bond must be in the Z-configuration in order to attack the oxime carbon for cyclization. The activation energy of this cyclization step is calculated as 14.1 kcal/mol for **Z-6** and 21.6 kcal/mol for **E-6**. After cyclization, the zwitterionic intermediate **INT1-M1** forms, and the cyanide eliminates to form a stable cation **INT2-M1**. The planar **INT2-M1** satisfies Hückel’s rules,^21−23^ with six electrons in the perpendicular p-orbitals of the five-membered ring, thus possessing aromaticity; the consequent stability prevents **INT2-M1** from reverting to **INT1-M1**. In the last step, **INT2-M1** loses the proton to form **7**.

**Figure 2:**
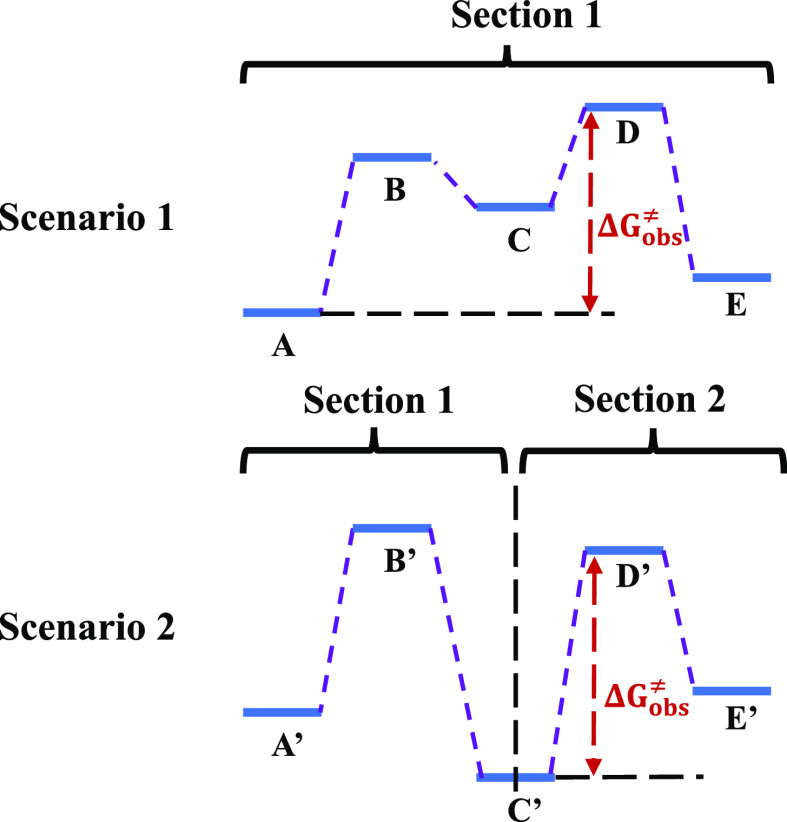
Illustration of Murdoch’s approach.

To determine the RDS, Murdoch’s approach,^24^ which divides a multistep reaction sequence into sections, is applied (Figure 2). The first section starts with the initial reactant(s) and terminates with the first intermediate that is more stable than the initial reactant(s). If there is no such intermediate, the whole reaction sequence consists of only one section (Scenario 1 in Figure 2). Otherwise, a second section is obtained in the same way by treating the terminating intermediate in preceding section as the new initial reactant (Scenario 2 in Figure 2). The procedure is repeated until all species involved belong to a section. The RDS is the step that directly results in the transition state with the highest free energy relative to the starting reactant(s) in the section containing the transition state. This relative free energy is also the “observed” reaction activation Gibbs free energy  that represents the kinetics observed experimentally.

In **M1**, the RDS of the reaction is determined as the initial cyclization step for both **M1**(E) and **M1**(Z), with Δ*G*_obs_^⧧^, calculated as the difference in Gibbs free energy between **TS1-M1** and **6**, equal to 21.7 kcal/mol for **M1**(Z) and 23.1 kcal/mol for **M1**(E). Because of its lower Δ*G*_obs_^⧧^, the reaction starting from Z-**6** proceeds faster, although **TS1-M1(Z)** is higher in absolute free energy.

### Mechanism **M2**: Nucleophilic Attack by the sp^2^-Nitrogen at the Ester Carbonyl Carbon

In our investigation, another reaction mechanism (Mechanism **M2**, shown in green in Scheme 4) has also been discovered for the generation of HCN along with a six-membered ring product **11**, which was not reported in the relevant experimental works.^6,7^ It offers less flexibility with respect to conformation as it applies only to the combination of **Z-6** with the E-imine. In this reaction mechanism, the ester carbonyl group is attacked by the sp^2^-nitrogen and forms a zwitterionic six-membered ring intermediate **INT1-M2** with a low activation barrier of 4.9 kcal/mol. In the next step, the ethoxide is directly transferred to the adjacent oxime carbon. While this type of ethoxide transfer is quite unusual, and we are not aware of a precedent in the literature, the outcome is identical to that of sequential ethoxide elimination and addition steps, which would represent the generally accepted mechanism. However, we have not been able to locate this more conventional stepwise process; only the concerted pathway is found. This can be attributed to the favorable spatial arrangement during the elimination of the ethoxide group attracted by the adjacent oxime group. The attacked oxime carbon thus turns from sp^2^ to sp^3^ hybridized, after which the cyanide group can be eliminated. Finally, HCN forms via proton transfer. In this mechanism, the ethoxide transfer step is the RDS instead of the cyclization step. We have calculated the Δ*G*_obs_^⧧^ of this reaction pathway (using Murdoch’s approach) to be 28.98 kcal/mol. The smaller Δ*G*_obs_^⧧^ values of both variants of **M1** thus support that the five-membered ring product **7** is likely to dominate, and **M1** plays a much larger role in HCN generation.

Nevertheless, the six-membered ring product **11** is isomeric with the five-membered ring product **7** proposed by McFarland et al.^6^ and the two would be expected to have similar spectroscopic data. Therefore, the six-membered ring product cannot be immediately ruled out. In order to help distinguish **7** and **11**, we have calculated the ^13^C NMR spectra of both structures using the GIAO method^15^ at the same level of theory as geometry optimization in Gaussian software. The predicted chemical shifts of **7** (Table 1) are overall closer to the experimental values reported earlier,^6^ supporting the idea that the 5-membered ring structure is the experimentally observed product.

**Table 1:** Three Lowest Field ^13^C Chemical Shifts for the Five-Membered Ring Product **7** and Six-Membered Ring Product **11**, the Experimental Chemical Shifts for the Cyclic Product and the Respective Mean Absolute Error of the Chemical Shifts for All the Carbon Atoms in **7** and **11**^a^

Experimental (ppm)	Five-membered ring **7** (ppm)	Six-membered ring **11** (ppm)
156.5	155.5	153.0
150.6	150.6	146.1
149.5	146.8	139.1
MAE for all carbon atoms (ppm)	2.8	3.9

a The full assignment is in the Supporting Information.

## Conclusion

We show that the proposed reaction pathway for HCN generation from Oxyma and DIC is strongly supported by our computational analysis and new details of the reaction mechanisms have been elucidated by DFT calculations. In particular, the important impact of oxime configuration on the reaction mechanism has been explored and found to lead to a difference in energy barriers of several kcal/mol. These results suggest that the formation of the Oxyma/DIC adduct **7** is faster than the cyclization and reversible, and the RDS is the initial cyclization step. From our DFT calculations, another reaction pathway has also been identified to generate HCN along with a six-membered ring product. The predominance of the mechanism involving the five-membered ring product can be justified on the basis of an energetic analysis by computing the “observed” activation energy Δ*G*_obs_^⧧^ and the comparison between the predicted and experimental ^13^C NMR. Moreover, our calculations can facilitate the derivation of other useful information, such as the kinetic isotope effects^25,26^ and infrared spectra predictions, which can be used to compare with corresponding experimental results for further verification of the identified reaction pathways. Our work provides theoretical support for the kinetic modeling^27^ of HCN formation in peptide synthesis and the design of a strategy for suppressing the generation of HCN when using Oxyma and DIC as amino acid activation reagent. An example of such a strategy is the QM-CAMD (quantum mechanical computer-aided molecular design) method for solvent design.^28^ Our findings pinpoint the most influential step in HCN formation from a kinetics perspective, alleviating the need to investigate the entire reaction landscape. The development of such a strategy and its application to the suppression of HCN formation in peptide synthesis is the subject of current work.^29^

## Supporting Information Available

Reaction schemes with energy profiles of Mechanism **M3**, Mechanism **M4**, proton transfer in Mechanism **M1** and Mechanism **M2**, summary of all the identified mechanisms, the computed electron density of the Oxyma/DIC adduct, the full data of the computed NMR spectra for the 5- and 6-membered ring products, the details of the conformer search of the most stable transition state **TS1-M1**, the computed energy profiles of the reactions of Oxyma and DTBC/TBEC, the investigation of the solvent effects on the kinetics of HCN formation, the computed geometries in Cartesian coordinates and electronic and Gibbs free energies for all the optimized structures (PDF)
