# Supplementary material for: Uncovering the Most Kinetically Influential Reaction Pathway Driving the Generation of HCN from Oxyma/DIC Adduct: A Theoretical Study
Source: Ind Eng Chem Res. 2023 Jan 5;62(2):874–80. doi: 10.1021/acs.iecr.2c03145 (PMC9853495; doi:10.1021/acs.iecr.2c03145)
Supplement: Supplementary file 1 — ie2c03145_si_001.pdf [file ie2c03145_si_001.pdf]

Supporting information.

Uncovering the most kinetically influential reaction pathway driving the generation of HCN from Oxyma/DIC adduct: a theoretical study.

Lingfeng Gui,[†] Claire S. Adjiman,[†] Amparo Galindo,[†] Fareed Bhasha Sayyed,[‡] Stanley P. Kolis[¶] and Alan Armstrong[*,§]

†Department of Chemical Engineering, The Sargent Centre for Process Systems Engineering and Institute for Molecular Science and Engineering, Imperial College London, London SW7 2AZ, UK

‡Synthetic Molecule Design and Development, Eli Lilly Services India Pvt Ltd, Devarabeesanahalli, Bengaluru-560103, India

¶Synthetic Molecule Design and Development, Eli Lilly and Company, Lilly Corporate Center, Indianapolis, Indiana 46285, United States

§Department of Chemistry and Institute for Molecular Science and Engineering, Imperial College London, Molecular Sciences Research Hub, White City Campus, London W12 0BZ, UK

E-mail: a.armstrong@imperial.ac.uk

Contents

Mechanism 3 (**M3**): a stepwise reaction via the nucleophilic attack of the sp^3-nitrogen 3

Mechanism 4 (**M4**): 5-endo-trig ring closure or an intramolecular substitution at a sp^2-hybridized carbon center? 4

Mechanism of proton transfer of **INT3-M2** and **INT2-M1** 5

Summary of the four identified mechanisms of the decomposition reaction of **6** 6

The computed electron density from total SCF density (isoval=0.0004, mapped with ESP) for Oxyma/DIC adduct **6** 7

The computed NMR spectra for the 5- and 6-membered ring products 8

The Conformer search of the most stable transition states 11

Exploring the steric effects of the alkyl groups bonded to the nitrogen atoms in the carbodiimide: Di-*tert*-butylcarbodiimide (DTBC) and *Tert*-butylethylcarbodiimide (TBEC) 12

Solvent effects on the kinetics of the HCN formation 16

The total electronic energies, thermal corrections to Gibbs free energy and atomic coordinates of the reactant, intermediates, and transition state structures in the mechanism of the addition reaction of DIC and E-Oxyma (Table S3-S8) 18

The total electronic energies, thermal corrections to Gibbs free energy and atomic coordinates of the reactant, intermediates, and transition state structures in the mechanism of the addition reaction of DIC and Z-Oxyma (Table S9-S12) 24

The total electronic energies, thermal corrections to Gibbs free energy and atomic

coordinates of the reactant, intermediates, and transition state structures in Mechanism **1** (E) (Table S13-S17) 28

The total electronic energies, thermal corrections to Gibbs free energy and atomic coordinates of the reactant, intermediates, and transition state structures in Mechanism **1** (Z) (Table S18-S22) 35

The total electronic energies, thermal corrections to Gibbs free energy and atomic coordinates of the reactant, intermediates, and transition state structures in Mechanism **2** (Table S23-S30) 42

The total electronic energies, thermal corrections to Gibbs free energy and atomic coordinates of the reactant, intermediates, and transition state structures in Mechanism **3** (Table S31-S34) 52

The total electronic energies, thermal corrections to Gibbs free energy and atomic coordinates of the reactant, intermediates, and transition state structures in Mechanism **4** (Table S35-S36) 58

The total electronic energies, thermal corrections to Gibbs free energy and atomic coordinates of the most stable conformers of **6** (Table S37-S38) 61

The total electronic energies, thermal corrections to Gibbs free energy and atomic coordinates of the transition state structures of Oxyma ion isomerization (Table S39-S40) 64

The total electronic energies, thermal corrections to Gibbs free energy and atomic coordinates of the conformers of **7** (Table S41-S48) 66

The total electronic energies, thermal corrections to Gibbs free energy and atomic coordinates of **11** (Table S49-S54) 76

Mechanism 3 (**M3**): a stepwise reaction via the nucleophilic attack of the sp^3-nitrogen

M3 (Scheme S1) is a stepwise reaction where the nucleophilic sp^3-nitrogen attacks the oxime carbon in **6** to form a zwitterionic intermediate **INT-M3**, following which the cyanide is eliminated. Then the proton on the positively charged nitrogen is removed by either the cyanide or the DMF solvent molecule. In the latter case, the protonated DMF can also transfer the proton to the cyanide. The cyclization step corresponds to a 5-endo-trig process in Baldwin's classification, a ring-closure mode that is usually disfavored. It was also found that **INT-M3** can react reversely back into **6** in the opposite oxime configuration, which essentially acts as an isomerization pathway.

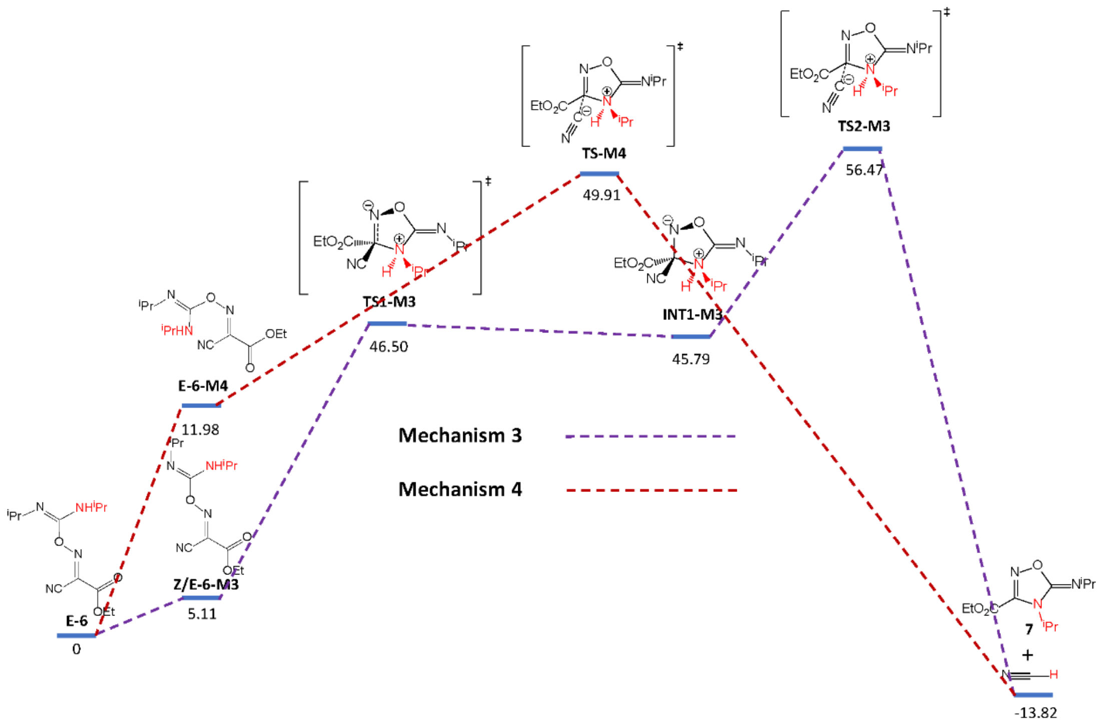

Scheme S1. Schematic representation of Mechanism 3 and Mechanism 4

Mechanism 4 (**M4**): 5-endo-trig ring closure or an intramolecular substitution at a sp^2-hybridized carbon center?

In **M4**, **6** proceeds via a similar nucleophilic attack as in **M3** but directly results into **7** without an intermediate. When the cyanide eliminates, the proton binds to it at the same time to form HCN. Therefore, **M4** is a concerted reaction. Only **E-6** is reactive since the antiperiplanar electron pair on the oxime nitrogen facilitates the direct elimination in **E-6**. **M4** seems to be a more unusual intramolecular substitution reaction that directly occurs at an sp^2-hybridized carbon center. However, in the IRC profile (Figure S1) of the transition structure **TS-M4**, **6** first goes through a point (circled in Figure S1) whose gradient is very close to zero. The structure corresponding to it resembles **TS1-M3** in **M3**, where the double bond is partially broken. Although it is not a real stationary point on the potential energy surface, it can be viewed as a "hidden intermediate", and the favorable antiperiplanar electron pair contributes to a barrierless elimination. Therefore, the concerted reaction is essentially still a 5-endo-trig ring closure reaction.

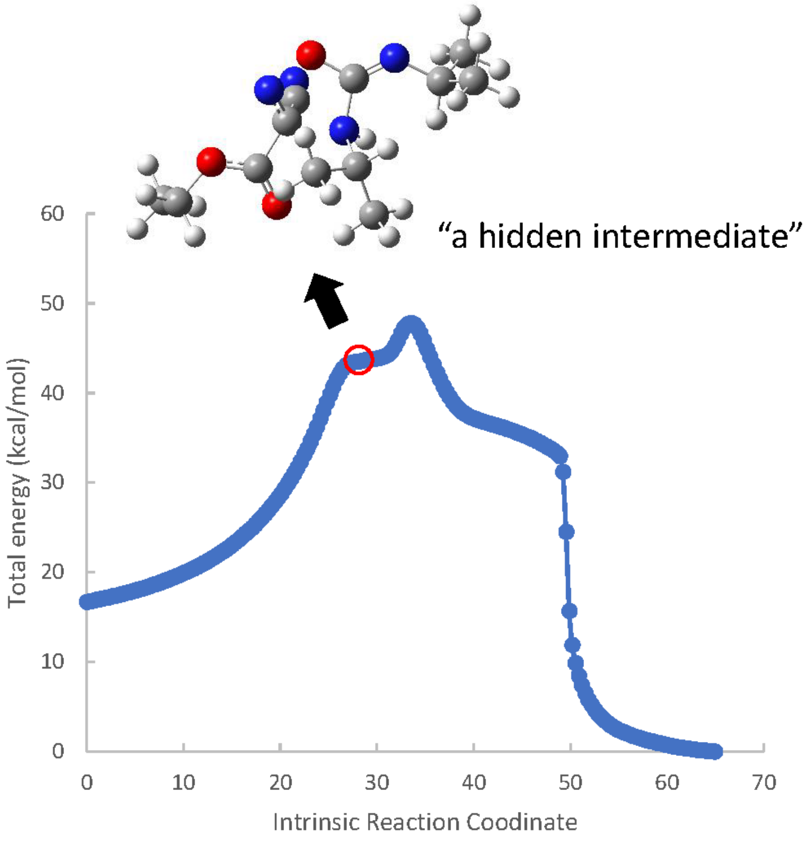

Figure S1. The IRC profile of the transition structure **TS-M4**

Mechanism of proton transfer of **INT3-M2** and **INT2-M1**

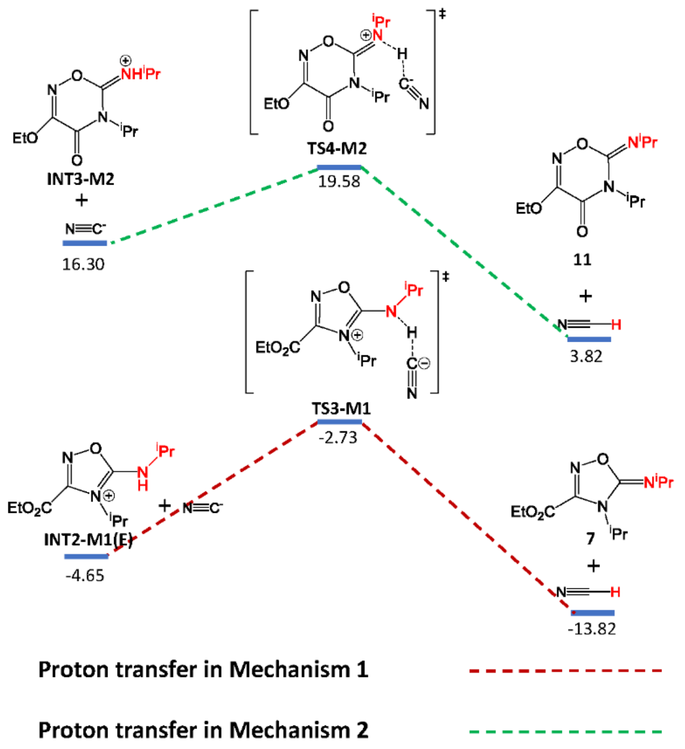

Scheme S2. Schematic representation of the mechanism of proton transfer of **INT3-M2** and **INT2-M1**

Summary of the four identified mechanisms of the decomposition reaction of **6**

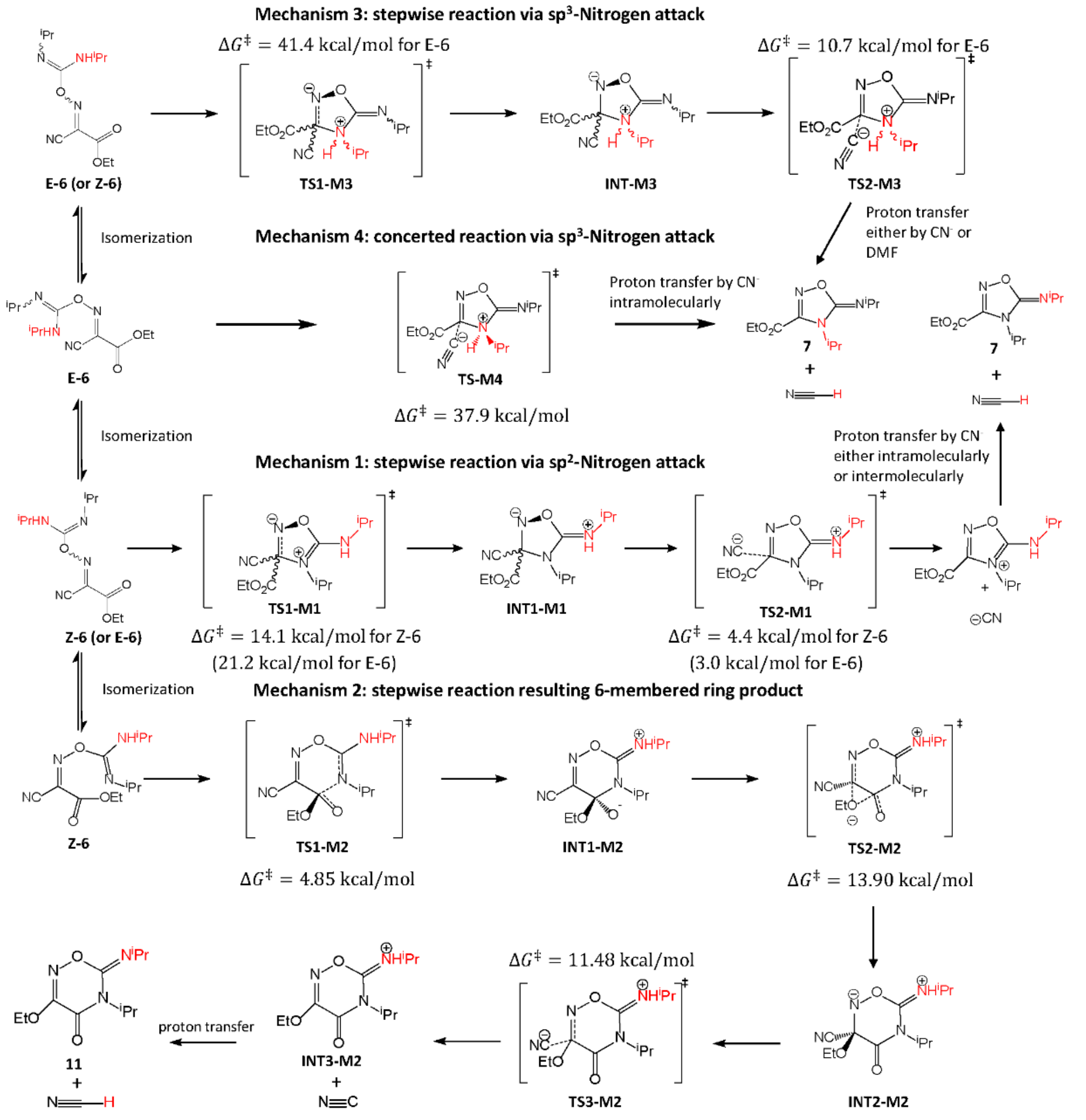

Scheme S3. Schematic representation of the four identified mechanisms of the decomposition reaction of **6**

The computed electron density from total SCF density (isoval=0.0004, mapped with ESP) for Oxyma/DIC adduct **6**

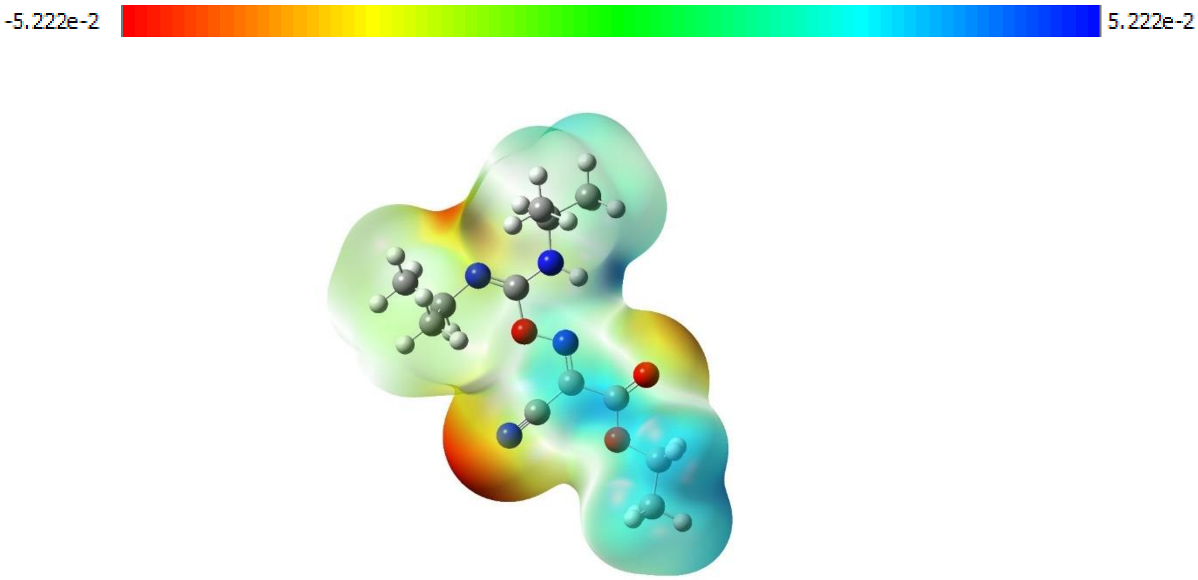

Figure S2. The computed electron density from total SCF density (isoval=0.0004, mapped with ESP) for Oxyma/DIC adduct **6**

The computed NMR spectra for the 5- and 6-membered ring products

Gauge-Independent Atomic Orbital (GIAO) method, B3LYP/6-31+g(d), TMS as reference (NH3 as reference for nitrogen), no scaling parameter is used.

The chemical shifts are calculated by taking the Boltzmann average over all the conformers. The conformers are identified by first doing grid search in Gaussview GMMX utility and then optimizing with B3LYP/6-31+g(d) in Gaussian 16.

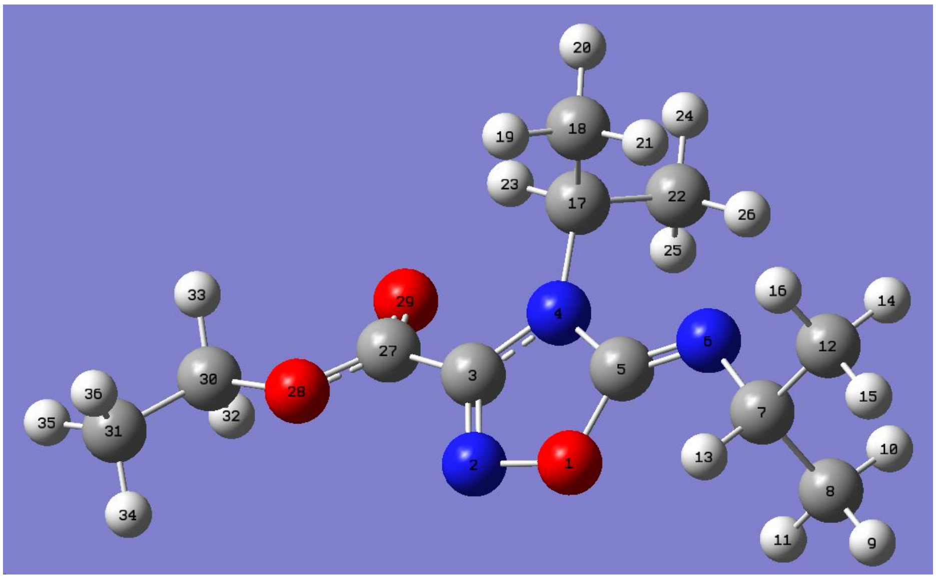

Figure S3. Labeled structure of **7**

Table S1.1. Predicted carbon chemical shifts for the five-membered ring product **7**

Atom	Chemical shift (ppm)	Experimental	AE
C27	155.5	156.5	1.0
C3	150.6	150.6	0.0
C5	146.8	149.5	2.7
C30	68.8	63.3	5.5
C17	53.8	48	5.8
C7	52.7	46.9	5.8
C8	27.5	24.2	3.3
C12	24.7	24.2	0.5
C18	19.6	18.2	1.4
C22	19.8	18.2	1.6
C31	16.2	13.4	2.8
MAE			2.8

Table S1.2. Predicted hydrogen chemical shifts for the five-membered ring product **7**

Atom	Five-membered ring	Experimental
H23	4.4	4.7
H32,H33	4.5	4.5
H13	3.9	3.8
H26,H21,H19,H20,H25,H24	1.4	1.5
H36,H34,H35	1.4	1.4
H11,H16,H15,H10,H9,H14	1.2	1.1
MAE	0.1	

Table S1.3. Predicted nitrogen chemical shifts for the five-membered ring product **7**

Atom	Five-membered ring	Experimental
N2	335.3	NA
N6	188.3	178.6
N4	149.8	126.4
MAE	16.6	

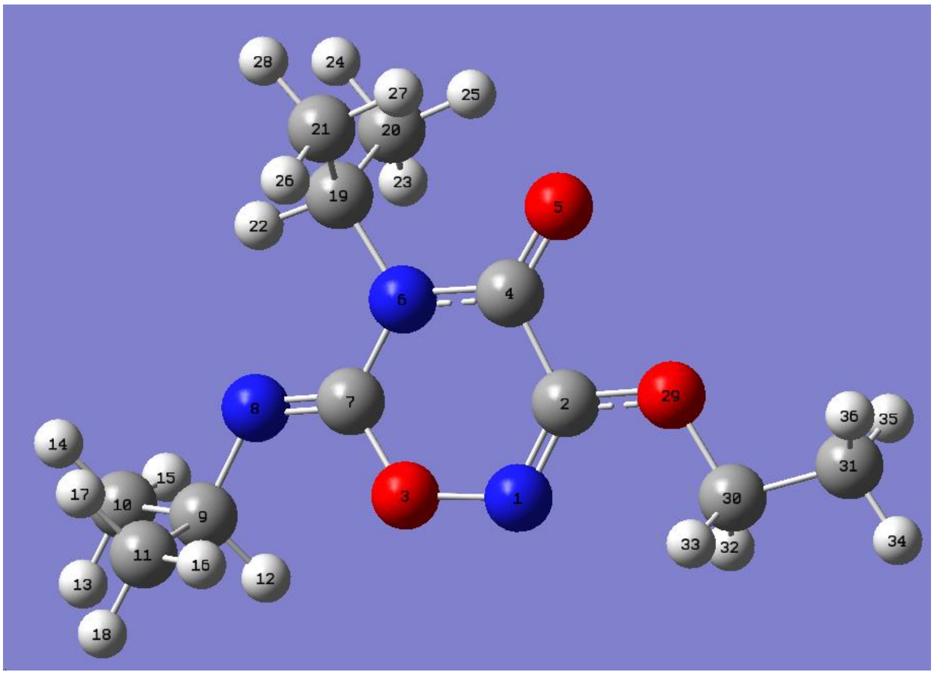

Figure S4. Labeled structure of **11** (hydrogen atoms not showing)

Table S2.1. Predicted carbon chemical shifts for the six-membered ring product **11**

Atom	Chemical shift (ppm)	Experimental	AE
C2	153.0	156.5	3.5
C4	146.1	150.6	4.5
C7	139.1	149.5	10.4
C30	69.0	63.3	5.7
C19	53.3	48	5.3
C9	52.0	46.9	5.1
C10	26.9	24.2	2.7
C11	24.0	24.2	0.2
C21	19.8	18.2	1.6
C20	18.8	18.2	0.6
C31	16.2	13.4	2.8
MAE			3.9

Table S2.2. Predicted hydrogen chemical shifts for the six-membered ring product **11**

Atom	Six-membered ring	Experimental
H22	4.9	4.7
H33,H32	4.2	4.5
H12	4.0	3.8
H27,H25,H26,H23,H28,H24	1.4	1.5
H36,H35,H34	1.4	1.4
H15,H14,H16,H18,H13,H17	1.2	1.1
MAE	0.1	

Table S2.3. Predicted nitrogen chemical shifts for the six-membered ring product **11**

Atom	Five-membered ring	Experimental
N1	328.6	NA
N8	222.4	178.6
N6	177.5	126.4
MAE	47.5	

The Conformer search of the most stable transition states

As discussed in Section 3.2 of the paper, the most kinetically influential pathway of HCN generation can be found via a systematic conformer search for the most stable stereoisomer of **TS1-M1**. However, it is computationally more difficult to perform a conformer search of a transition state structure. According to Hammond's postulate, the geometry of **TS1-M1** should resemble that of **INT1-M1** as they have closer energies. Therefore, the strategy taken here is that a relaxed multidimensional potential energy surface scan is carried out first to obtain all the stereoisomers of **INT1-M1**, and then the corresponding transition states **TS1-M1** are derived from the stereoisomers of **INT1-M1**. The stereoisomers of **INT1-M1** have been first classified into four groups (Figure 1 in the paper) based on their imine and oxime bond configurations. In each group, the imine and oxime bond configurations are fixed, and the two N-isopropyl bonds were scanned by rotating each of them with a step size of 30° to generate 144 combinations (grid points). The minima on the two-dimensional potential energy surface were then optimized with B3LYP-D3/6-31+G(d). The corresponding transition states were then derived from each conformer of **INT1-M1** by scanning the potential energy surface over the C(oxime)-N(amine) bond distance and optimizing the identified saddle point.

Exploring the steric effects of the alkyl groups bonded to the nitrogen atoms in the carbodiimide: Di-*tert*-butylcarbodiimide (DTBC) and *Tert*-butylethylcarbodiimide (TBEC)

It was suggested by Manne et al. that the steric hindrance caused by the side chains attached to the carbodiimide greatly affects the amount of HCN formed. They found that the carbodiimide with two tertiary carbons attached, i.e., DTBC, does not produce HCN as it does not form an adduct with Oxyma. The asymmetrical carbodiimide, TBEC, forms the linear product with Oxyma but goes through a different cyclisation path from that for DIC and generates a six-membered ring oxadiazine product without HCN formed.

To investigate further the effects of the steric hindrance on the formation of HCN, we study the addition of Oxyma and DTBC/TBEC and the cyclisation of the adduct produced and compare the computed "observed" activation free energies with those for DIC.

In the case of DTBC (Animation S1 and S2), although the addition reaction requires only 11.5 kcal/mol and thus proceeds fast, the cyclisation reaction must overcome a free energy barrier of 27.86 kcal/mol (Scheme S4). Without sufficient energy to activate the adduct at room temperature, the reaction is thermodynamically driven towards the reactants (DTBC and Oxyma), which explains why there is no adduct observed and no Oxyma consumed when using DTBC to react with Oxyma.

For the asymmetrical carbodiimide TBEC (Animation S3, S4, S5 and S6), the activation Gibbs free energy for the addition reaction with Oxyma and the subsequent cyclisation is similar regardless of whether it is the primary or tertiary alkyl group that is attached to the nitrogen attacking the oxime carbon (Scheme S5 and S6). The activation Gibbs free energy required for the subsequent cyclisation is lower than that for DTBC but higher than that for DIC (23.07 kcal/mol). This may explain why Manne et al. observed a significant amount of oxadiazole, the indicator for HCN produced only after four days of reaction with Oxyma.

Our results are consistent with the experimental observations reported by Manne et al. and provide a possible explanation from a computational point of view as to why different amounts of HCN are formed when using carbodiimide with different types of side chains.

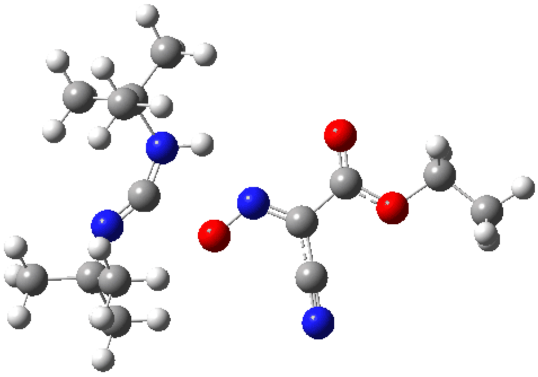

Animation S1. The vibrational mode corresponding to the addition of DTBC and Oxyma

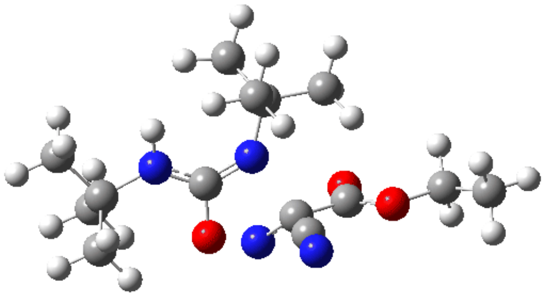

Animation S2. The vibrational mode corresponding to the cyclisation of DTBC/Oxyma adduct

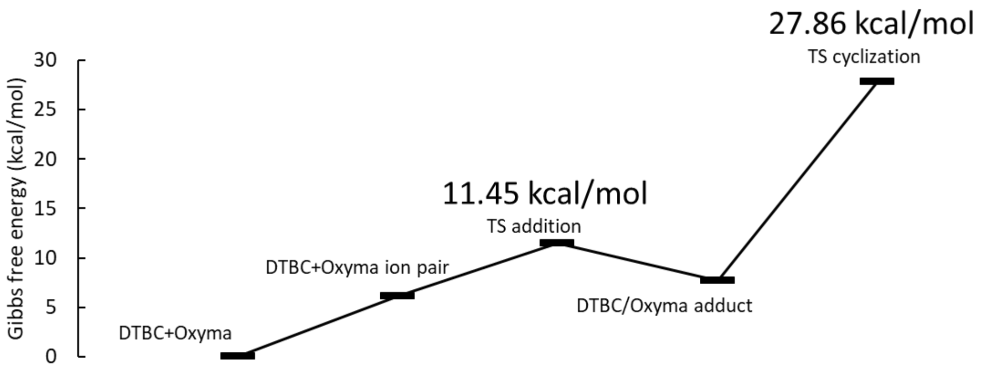

Scheme S4. The free energy profile of the reaction of DTBC and Oxyma

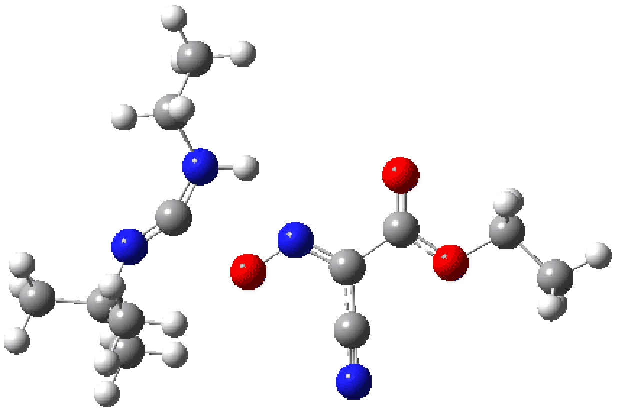

Animation S3. The vibrational mode corresponding to the addition of TBEC and Oxyma

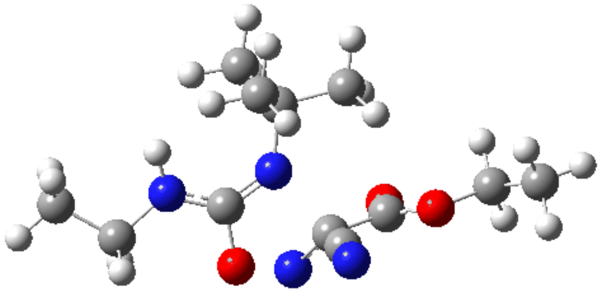

Animation S4. The vibrational mode corresponding to the cyclisation of TBEC/Oxyma adduct (via the attack of the nitrogen bonded to tert-butyl group)

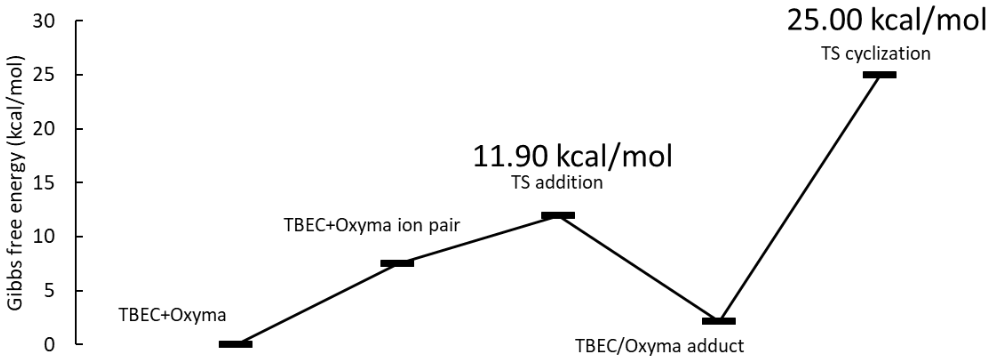

Scheme S5. The free energy profile of the reaction of TBEC and Oxyma (cyclisation via the attack of the nitrogen bonded to tert-butyl group)

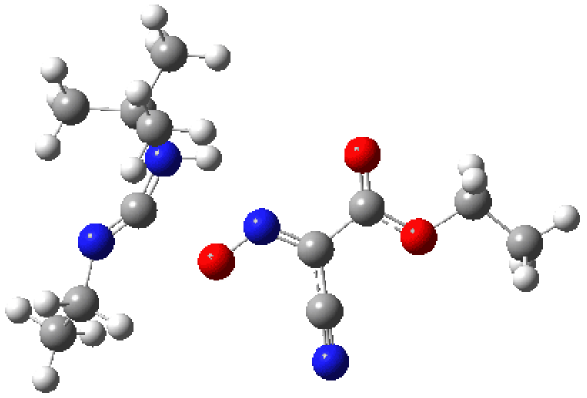

Animation S5. The vibrational mode corresponding to the addition of TBEC and Oxyma

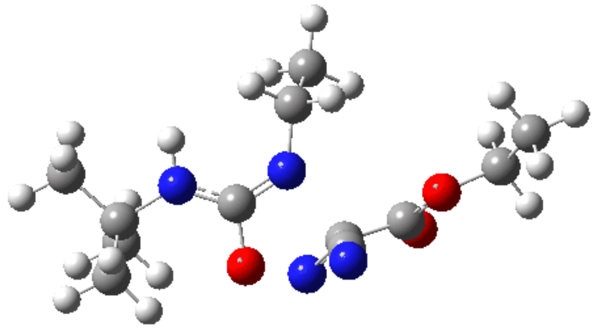

Animation S6. The vibrational mode corresponding to the cyclisation of TBEC/Oxyma adduct (via the attack of the nitrogen bonded to ethyl group)

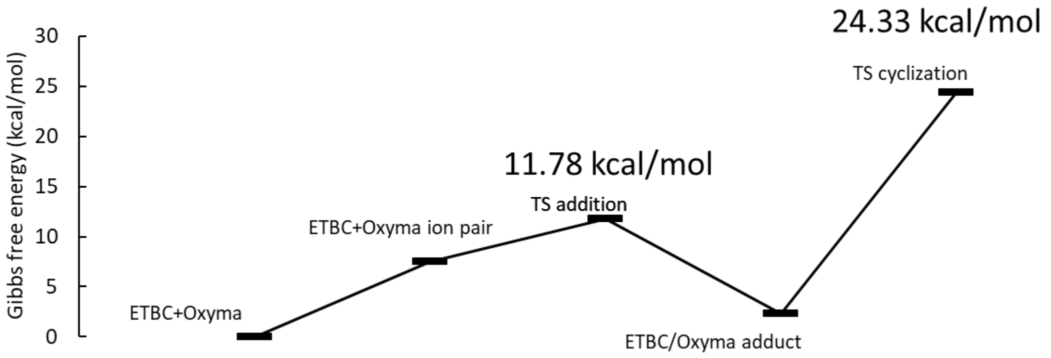

Scheme S6. The free energy profile of the reaction of TBEC and Oxyma (cyclisation via the attack of the nitrogen bonded to ethyl group)

Solvent effects on the kinetics of the HCN formation

The “observed” rate constants for the decomposition reaction of the Oxyma/DIC adduct are calculated using transition state theory in vacuo and in the solvents shown in the figure below. (The activation Gibbs free energies are calculated using the thermodynamic cycle method with G3MP2 as the level of theory for calculating the vacuum phase component and M062X/6-31+g(d)/SMD as the level of theory for the solvation energy component.)

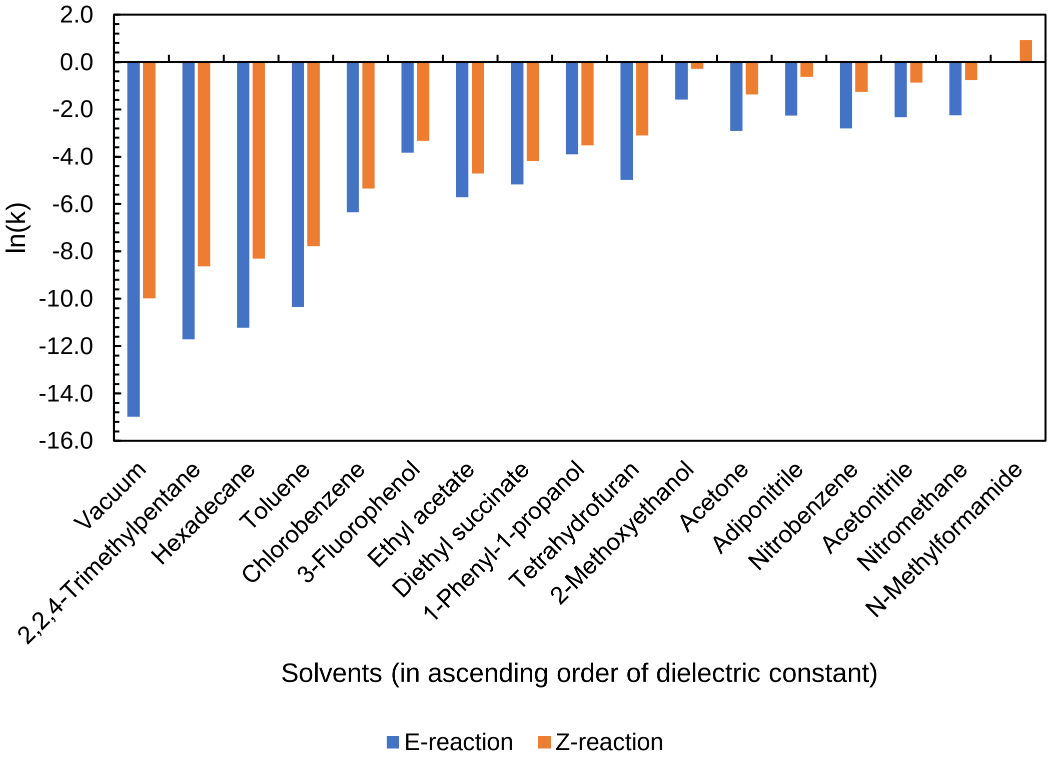

Figure S5. Logarithms of the calculated "observed" rate constants for the decomposition reaction starting from E- and Z- conformer

Table S3. Calculated "observed" rate constants for the decomposition reaction starting from E- and Z-conformer (original data for Figure S5)

Solvent	Eps	Computed rate constant/s^{-1} (E-reaction)	Computed rate constant/s^{-1} (Z-reaction)
Vacuum	1	3.11E-07	4.62E-05
2,2,4-Trimethylpentane	1.94	8.24E-06	1.79E-04
Hexadecane	2.04	1.33E-05	2.47E-04
Toluene	2.37	3.19E-05	4.16E-04
Chlorobenzene	5.70	1.75E-03	4.80E-03
3-Fluorophenol	5.90	2.16E-02	3.60E-02
Ethyl acetate	5.99	3.33E-03	9.05E-03
Diethyl succinate	6.10	5.70E-03	1.53E-02
1-Phenyl-1-propanol	6.68	2.02E-02	2.97E-02
Tetrahydrofuran	7.43	6.84E-03	4.52E-02
2-Methoxyethanol	17.20	2.05E-01	7.51E-01
Acetone	20.49	5.44E-02	2.55E-01
Adiponitrile	32.12	1.05E-01	5.31E-01
Nitrobenzene	34.81	6.04E-02	2.82E-01
Acetonitrile	35.69	9.70E-02	4.21E-01
Nitromethane	36.56	1.05E-01	4.70E-01
N-Methylformamide	181.56	1.00E+00	2.54E+00

The total electronic energies, thermal corrections to Gibbs free energy and atomic coordinates of the reactant, intermediates, and transition state structures in the mechanism of the addition reaction of DIC and E-Oxyma (Table S3-S8)

Table S4: total electronic energy, thermal correction to Gibbs free energy and atomic coordinates of DIC

Total electronic energy (Hartree): -384.707917112

Thermal correction to Gibbs Free Energy (Hartree): 0.166232

Atomic coordinates:

7	-1.17230900	-0.56833500	-0.35411300
6	0.00000000	-0.48831600	0.00000000
7	1.17230900	-0.56833500	0.35411300
6	2.29776400	0.22780600	-0.19305700
6	2.87558600	1.09636100	0.92570000
1	3.71691700	1.68743800	0.54416900
1	3.23778100	0.47019300	1.75090000
1	2.11938000	1.78574300	1.31916900
6	3.33992000	-0.73407000	-0.76611100
1	1.92784800	0.87730700	-0.99741800
1	3.71770300	-1.40367300	0.01696200
1	4.18543300	-0.16864000	-1.17619200
1	2.91171500	-1.34565800	-1.56890000
6	-2.29776400	0.22780600	0.19305700
6	-2.87558500	1.09636200	-0.92570000
1	-2.11937900	1.78574300	-1.31916900
1	-3.71691700	1.68743800	-0.54416900
1	-3.23778100	0.47019400	-1.75090000
6	-3.33992100	-0.73407000	0.76611000
1	-1.92784900	0.87730700	0.99741800
1	-4.18543400	-0.16864000	1.17619100
1	-2.91171600	-1.34565800	1.56890000
1	-3.71770300	-1.40367300	-0.01696200

Table S5: total electronic energy, thermal correction to Gibbs free energy and atomic coordinates of protonated DIC

Total electronic energy (Hartree): -385.147133885

Thermal correction to Gibbs Free Energy (Hartree): 0.178456

Atomic coordinates:

7	-1.23430100	-0.67182800	-0.30011400
6	0.02302600	-0.47101800	-0.16628800
7	1.16161700	-0.31339100	0.06892800
6	2.48114300	0.20918800	-0.30515100
6	2.82587700	1.34791300	0.65589600
1	3.79968800	1.75879500	0.36900700
1	2.88878100	0.98096100	1.68631200
1	2.08057000	2.14858200	0.60489100
6	3.48469400	-0.94214300	-0.25340500
1	2.38900400	0.58982000	-1.32717600
1	3.56566100	-1.34080700	0.76402300
1	4.46489200	-0.56328300	-0.56174300
1	3.19489800	-1.75090400	-0.93257000
6	-2.30860600	0.27757900	0.16503900
6	-3.04437700	0.81572200	-1.05814600
1	-2.35929300	1.34312800	-1.73060400
1	-3.82173800	1.51576800	-0.73214100
1	-3.52575600	-0.00057000	-1.61062400
6	-3.21362200	-0.47081700	1.13848600
1	-1.78534600	1.08710900	0.68023700
1	-3.99555100	0.20765800	1.49731300
1	-2.64756500	-0.83848000	2.00105500
1	-3.69719300	-1.32124600	0.64194900
1	-1.53106000	-1.51855300	-0.79020900

Table S6: total electronic energy, thermal correction to Gibbs free energy and atomic coordinates of **E-10**

Total electronic energy (Hartree): -913.996417433

Thermal correction to Gibbs Free Energy (Hartree): 0.263897

Atomic coordinates:

8	-0.50241200	-0.94389900	-0.08865900
7	0.49516400	-0.13735400	-0.12548400
6	1.71773600	-0.62396800	-0.01927200
7	-1.74189900	1.35995900	-0.44341000
6	-2.29042000	0.19085900	-0.38592800
7	-3.21303700	-0.57226800	-0.47964200

6	-3.61380300	-1.83385500	0.15987900
6	-4.77945600	-1.55098600	1.11070600
1	-5.07898700	-2.48363700	1.60222100
1	-5.64108100	-1.15512800	0.55995500
1	-4.49234200	-0.82836100	1.88264600
6	-3.97819500	-2.84339300	-0.92753200
1	-2.75295900	-2.19819300	0.73068000
1	-4.82654400	-2.48300800	-1.52194800
1	-4.25782800	-3.79526600	-0.46192500
1	-3.12993300	-3.02177800	-1.59788100
6	-2.42196000	2.60842500	0.01908300
6	-2.04712200	2.89190100	1.47328700
1	-2.34480300	2.06251700	2.12466500
1	-2.55473000	3.80138300	1.81530900
1	-0.96482500	3.04303400	1.56966000
6	-2.02250600	3.73665400	-0.92881500
1	-3.49541800	2.41490900	-0.06219000
1	-2.51861900	4.66443800	-0.62280600
1	-2.31414600	3.50808100	-1.95982000
1	-0.93800200	3.90176900	-0.89830400
6	2.84409000	0.33831900	-0.07490400
8	4.02181100	-0.29841400	0.04453900
8	2.72375600	1.54484300	-0.21095500
6	5.22640000	0.52207600	0.00148300
6	6.41106600	-0.40828200	0.15182900
1	5.17356100	1.25414700	0.81343900
1	5.24376100	1.05832400	-0.95259600
1	6.37087500	-0.94642600	1.10575800
1	7.33564500	0.18059100	0.12632100
1	6.44451500	-1.13879600	-0.66453400
6	1.95016200	-2.02421100	0.14393100
1	-0.71226000	1.36740000	-0.49537400
7	2.10733300	-3.17140500	0.27780100

Table S7: total electronic energy, thermal correction to Gibbs free energy and atomic coordinates of **E-6**

Total electronic energy (Hartree): -914.017936434

Thermal correction to Gibbs Free Energy (Hartree): 0.269229

Atomic coordinates:

8	-0.54604500	-0.52561800	-0.04466600
7	0.47829100	0.36812000	-0.03670000
6	1.63722300	-0.19805600	0.00141700
7	-1.89337600	1.38493200	-0.28471400
6	-1.85632200	0.04573300	-0.08792400
7	-2.85186500	-0.73620300	0.01212200
6	-2.68955500	-2.19219200	0.13917800
6	-3.90905100	-2.74635900	0.88022600
1	-3.82018600	-3.83211100	1.00906900
1	-4.82876900	-2.53849200	0.31765600
1	-4.00451000	-2.28909100	1.87273500
6	-2.54363200	-2.83352300	-1.24859400
1	-1.79235800	-2.43362900	0.72626800
1	-3.41971200	-2.60532500	-1.86940800
1	-2.45889900	-3.92381300	-1.15897200
1	-1.64981500	-2.46228700	-1.76361000
6	-3.14384400	2.15396900	-0.16640400
6	-3.52966100	2.36858200	1.30171200
1	-3.64009200	1.41060600	1.82191100
1	-4.48252700	2.90770600	1.37028400
1	-2.76113500	2.95958700	1.81658200
6	-2.96648300	3.47623700	-0.91337900
1	-3.92119400	1.56244100	-0.65992900
1	-3.89121400	4.06194600	-0.86330700
1	-2.72105400	3.30383200	-1.96787500
1	-2.16242200	4.07373400	-0.46297500
6	2.83595100	0.71810300	0.01708200
8	3.95641200	0.00247400	0.06417700
8	2.75512000	1.92854800	-0.00975200
6	5.22247700	0.74357300	0.08116900
6	6.33788100	-0.27537100	0.14236300
1	5.21223400	1.40308500	0.95377800
1	5.26448600	1.35357900	-0.82590400
1	6.26646900	-0.88476300	1.05012200

1	7.29850700	0.25271800	0.15552400
1	6.31863600	-0.93525600	-0.73212400
6	1.81036300	-1.62441300	0.03001900
1	-1.03035300	1.89323900	-0.12660800
7	1.93379700	-2.77951200	0.05179300

Table S8: total electronic energy, thermal correction to Gibbs free energy and atomic coordinates of E-Oxyma

Total electronic energy (Hartree): -529.284924584

Thermal correction to Gibbs Free Energy (Hartree): 0.078451

Atomic coordinates:

8	-3.31149600	-0.16076700	-0.00000200
7	-2.14252100	-0.85807600	-0.00000100
6	-1.09563500	-0.10387900	0.00000000
6	0.24281000	-0.79995900	0.00000100
8	1.22490300	0.09875100	-0.00000100
8	0.37411900	-2.00625700	0.00000300
6	2.59945300	-0.41281600	-0.00000000
6	3.52329600	0.78435800	-0.00000200
1	2.72556100	-1.03555600	-0.89059600
1	2.72556200	-1.03555500	0.89059600
1	3.36898600	1.40198600	-0.89180900
1	4.56098300	0.43075800	-0.00000300
1	3.36898700	1.40198700	0.89180500
6	-1.16554300	1.33216900	0.00000100
7	-1.23921600	2.49175400	0.00000200
1	-4.00440800	-0.85242000	-0.00000300

Table S9: total electronic energy, thermal correction to Gibbs free energy and atomic coordinates of deprotonated E-Oxyma

Total electronic energy (Hartree): -528.835056970

Thermal correction to Gibbs Free Energy (Hartree): 0.065960

Atomic coordinates:

8	3.36949300	-0.29572800	0.00000100
7	2.23990300	-0.87549600	0.00000100
6	1.13072700	-0.10649900	-0.00000000
6	-0.17546500	-0.77478200	-0.00000000

8	-1.18753400	0.12800100	-0.00000000
8	-0.36391100	-1.98511200	-0.00000100
6	-2.53965500	-0.40129400	-0.00000100
6	-3.48918800	0.77931500	0.00000200
1	-2.67173200	-1.02849200	0.88800600
1	-2.67173200	-1.02848900	-0.88800900
1	-3.34399800	1.40162500	0.89066800
1	-4.52286800	0.41293800	0.00000100
1	-3.34399800	1.40162800	-0.89066300
6	1.22396600	1.31339200	-0.00000100
7	1.34690200	2.47588300	-0.00000100

The total electronic energies, thermal corrections to Gibbs free energy and atomic coordinates of the reactant, intermediates, and transition state structures in the mechanism of the addition reaction of DIC and Z-Oxyma (Table S9-S12)

Table S10: total electronic energy, thermal correction to Gibbs free energy and atomic coordinates of **Z-10**

Total electronic energy (Hartree): -913.990053716

Thermal correction to Gibbs Free Energy (Hartree): 0.264902

Atomic coordinates:

8	-0.17259900	-0.54778200	-0.15016500
7	0.45377800	0.56056300	-0.19710300
6	1.78045700	0.60378600	-0.12797100
7	-2.17825500	1.18518500	-0.41464200
6	-2.31580600	-0.09606500	-0.38716900
7	-2.89519800	-1.14009000	-0.49268100
6	-2.82874300	-2.46800200	0.13625700
6	-3.99226900	-2.60166500	1.12169400
1	-3.94457500	-3.58468300	1.60379000
1	-4.95307200	-2.51560300	0.60042100
1	-3.94212600	-1.83090800	1.89875700
6	-2.86278100	-3.53343500	-0.95779100
1	-1.87812400	-2.52120000	0.67755800
1	-3.80156300	-3.47938200	-1.52221200
1	-2.78729800	-4.52656300	-0.50071900
1	-2.02608700	-3.40852300	-1.65418100
6	-3.21366000	2.13553900	0.09828100
6	-2.89647400	2.50866500	1.54565000
1	-2.88345900	1.62044600	2.18721700
1	-3.65902900	3.19922000	1.92421100
1	-1.91957900	3.00346900	1.61073400
6	-3.24304600	3.34330800	-0.83482600
1	-4.16614300	1.60036700	0.04714500
1	-4.00451600	4.05211900	-0.49090000
1	-3.48245200	3.04405500	-1.86116000
1	-2.27281000	3.85589600	-0.83460100
6	2.70293600	-0.54938200	0.01911900
8	3.98352600	-0.12454600	0.06147500

8	2.37006500	-1.71934900	0.09466500
6	5.01197400	-1.14570000	0.20815700
6	6.35019000	-0.43798900	0.23629100
1	4.93304700	-1.83982400	-0.63477500
1	4.82112900	-1.69852000	1.13362900
1	6.52262500	0.11611400	-0.69349800
1	7.14836900	-1.18155000	0.34799900
1	6.40878800	0.26032100	1.07902700
6	2.31281900	1.92702100	-0.20423800
1	-1.20386100	1.52636500	-0.48103600
7	2.73527500	3.01224300	-0.26785000

Table S11: total electronic energy, thermal correction to Gibbs free energy and atomic coordinates of **Z-6**

Total electronic energy (Hartree): -914.012641204

Thermal correction to Gibbs Free Energy (Hartree): 0.268461

Atomic coordinates:

8	0.34260200	0.15727200	-0.04423200
7	-0.26947100	-1.04702200	-0.03952600
6	-1.56246900	-1.00919500	0.00735600
7	2.30253900	-1.13233000	-0.25221100
6	1.77149000	0.10288800	-0.09116600
7	2.40953900	1.19902300	-0.02290400
6	1.72192500	2.49620900	0.05831100
6	2.61815800	3.46541200	0.83372500
1	2.13540100	4.44634800	0.92469400
1	3.57874200	3.59877400	0.31878300
1	2.82098800	3.08757000	1.84343700
6	1.42536800	3.02140100	-1.35386900
1	0.76898700	2.39973100	0.59536200
1	2.35515500	3.12245000	-1.92874200
1	0.94261100	4.00552800	-1.30256000
1	0.75745100	2.34042000	-1.89442600
6	3.74950400	-1.37880700	-0.13388600
6	4.19928400	-1.37627600	1.33179600
1	3.95039400	-0.42583000	1.81667300

1	5.28476300	-1.52049000	1.39732400
1	3.70950600	-2.18925100	1.88336100
6	4.06868800	-2.70229600	-0.82986100
1	4.24912100	-0.56222100	-0.66450200
1	5.14469500	-2.90298800	-0.77895600
1	3.76964500	-2.67587100	-1.88429000
1	3.54545300	-3.53544600	-0.34146200
6	-2.46092100	0.20596600	0.06681000
8	-3.73653200	-0.18223100	0.05773600
8	-2.06521600	1.35132900	0.11775000
6	-4.75310000	0.87302100	0.11597000
6	-6.10451100	0.19495100	0.09532000
1	-4.60378200	1.53023700	-0.74585000
1	-4.58865100	1.44650400	1.03305600
1	-6.24135200	-0.38413500	-0.82471700
1	-6.88637700	0.96214800	0.14035000
1	-6.22460100	-0.47167000	0.95655800
6	-2.16901900	-2.31414500	0.00592800
1	1.68980800	-1.91951500	-0.07073400
7	-2.65848700	-3.36796000	0.00578200

Table S12: total electronic energy, thermal correction to Gibbs free energy and atomic coordinates of Z-Oxyma

Total electronic energy (Hartree): -529.279567097

Thermal correction to Gibbs Free Energy (Hartree): 0.078450

Atomic coordinates:

8	2.76333600	-1.22896500	0.00000000
7	2.43462700	0.08491900	0.00000000
6	1.16775300	0.34877700	0.00000000
6	0.01057700	-0.62356500	0.00000000
8	-1.14148100	0.04913800	0.00000000
8	0.12884300	-1.83084800	-0.00000000
6	-2.37398700	-0.74446500	0.00000000
6	-3.53338100	0.22641100	-0.00000000
1	-2.36348900	-1.38025500	0.89028400
1	-2.36348900	-1.38025500	-0.89028400

1	-3.51553600	0.86285200	0.89174800
1	-4.47095200	-0.34184600	-0.00000000
1	-3.51553600	0.86285200	-0.89174900
6	0.88115100	1.75969200	0.00000000
7	0.64660100	2.89772900	0.00000000
1	3.74214200	-1.21758600	0.00000000

Table S13: total electronic energy, thermal correction to Gibbs free energy and atomic coordinates of deprotonated Z-Oxyma

Total electronic energy (Hartree): -528.827415823

Thermal correction to Gibbs Free Energy (Hartree): 0.065873

Atomic coordinates:

8	-2.85709800	-1.27718200	0.00000100
7	-2.51980000	-0.06020200	0.00000000
6	-1.20239800	0.27472800	-0.00000000
6	-0.04030400	-0.62768500	-0.00000000
8	1.12088600	0.08507900	0.00000000
8	-0.06344100	-1.84980700	-0.00000100
6	2.35620800	-0.67559900	-0.00000000
6	3.50076200	0.31754300	0.00000000
1	2.37556400	-1.31695400	-0.88776600
1	2.37556400	-1.31695500	0.88776500
1	3.46819100	0.95602800	-0.89052500
1	4.45334700	-0.22602800	-0.00000000
1	3.46819100	0.95602700	0.89052700
6	-0.98893200	1.68094400	-0.00000000
7	-0.83672100	2.84071300	-0.00000000

The total electronic energies, thermal corrections to Gibbs free energy and atomic coordinates of the reactant, intermediates, and transition state structures in Mechanism **1** (E) (Table S13-S17)

Table S14: total electronic energy, thermal correction to Gibbs free energy and atomic coordinates of **E-6-M1**

Total electronic energy (Hartree): -914.015894256

Thermal correction to Gibbs Free Energy (Hartree): 0.269053

Atomic coordinates:

8	-0.52004700	-0.43651600	0.53509600
7	0.44578300	0.35041700	-0.00193800
6	1.63289200	-0.09546300	0.24120700
7	-2.71316700	-0.91817200	0.32058900
6	-1.82691900	0.10905500	0.41649200
7	-1.97936000	1.36522900	0.51030100
6	-3.31893600	1.95152000	0.34639600
6	-3.32772200	3.33087700	1.00894700
1	-4.31875000	3.79298100	0.92279500
1	-2.59425200	3.99104600	0.52800900
1	-3.07427500	3.25462000	2.07336900
6	-3.68661800	2.04537900	-1.14247000
1	-4.07585900	1.33225300	0.85595400
1	-2.96009900	2.67440600	-1.67235100
1	-4.68191800	2.49041800	-1.26265300
1	-3.69518800	1.05714100	-1.61719500
6	-2.41132400	-2.24872500	-0.25844200
6	-3.59074100	-3.17148800	0.04673200
1	-3.76548700	-3.24483800	1.12646600
1	-3.38825700	-4.17576100	-0.34104500
1	-4.50853100	-2.80258100	-0.43067300
6	-2.12928800	-2.15296100	-1.76172200
1	-1.52342400	-2.63130600	0.25246900
1	-1.88433500	-3.14312600	-2.16516500
1	-1.28402700	-1.48463000	-1.96479700
1	-3.00880100	-1.76983500	-2.29453300
6	2.78165500	0.67929600	-0.35457000
8	3.93745100	0.10601500	-0.02537100

8	2.64307600	1.67655900	-1.03182700
6	5.16253900	0.73296100	-0.53141100
6	6.32671100	-0.09937900	-0.04289700
1	5.19580800	1.75838900	-0.15193900
1	5.09918600	0.75979800	-1.62323900
1	6.36085200	-0.12613000	1.05194500
1	7.25920200	0.34761400	-0.40690700
1	6.26462800	-1.12550200	-0.42214100
6	1.88575800	-1.26969900	1.03129500
1	-3.67965700	-0.62610400	0.21942800
7	2.07407300	-2.22112400	1.67128800

Table S15: total electronic energy, thermal correction to Gibbs free energy and atomic coordinates of **INT1-M1(E)**

Total electronic energy (Hartree): -913.999341855

Thermal correction to Gibbs Free Energy (Hartree): 0.274361

Atomic coordinates:

8	-1.13836300	-1.59735600	-0.12345900
7	0.36652700	-1.64297000	-0.36343600
6	0.77011100	-0.41136700	0.25562500
7	-2.74176200	0.02316800	0.06298700
6	-1.46985100	-0.34058900	0.03525000
7	-0.40744400	0.47317200	0.17754200
6	-0.51514500	1.93406500	0.47426000
6	0.69020200	2.45885800	1.25511000
1	0.52254000	3.52253400	1.45376500
1	1.62140700	2.37463300	0.68644000
1	0.80932400	1.95495300	2.21816600
6	-0.74920000	2.72208900	-0.81852500
1	-1.38810100	2.04121000	1.12936200
1	0.13430500	2.67058000	-1.46072900
1	-0.95161600	3.77068500	-0.57120500
1	-1.60783600	2.33326200	-1.37739200
6	-3.88689400	-0.87469300	-0.21394400
6	-4.99133700	-0.57380500	0.79880200
1	-4.63719800	-0.72720500	1.82447400

1	-5.84574200	-1.23617600	0.62199900
1	-5.33769500	0.46325500	0.69906400
6	-4.35102900	-0.69468700	-1.66135900
1	-3.52823200	-1.89598100	-0.06657600
1	-5.18896700	-1.36978000	-1.87094700
1	-3.54121900	-0.91905300	-2.36478800
1	-4.68672700	0.33586800	-1.83236300
6	1.97461900	0.07381300	-0.58290400
8	3.08586700	-0.53770900	-0.18635500
8	1.87671400	0.84351100	-1.51527900
6	4.30375500	-0.28117900	-0.96075300
6	5.41578400	-1.08881600	-0.32950500
1	4.50211800	0.79418100	-0.92882500
1	4.11218000	-0.57622000	-1.99670700
1	5.57819000	-0.78914200	0.71194400
1	6.34340100	-0.91519200	-0.88761500
1	5.19013900	-2.16068000	-0.35922100
6	1.15039700	-0.59790400	1.72553600
1	-2.95872200	1.00706800	0.18684600
7	1.38499800	-0.89638100	2.82569300

Table S16: total electronic energy, thermal correction to Gibbs free energy and atomic coordinates of **INT2-M1(E)**

Total electronic energy (Hartree): -821.045059528

Thermal correction to Gibbs Free Energy (Hartree): 0.270033

Atomic coordinates:

8	-1.02955500	-1.57048800	-0.19283100
7	0.39250900	-1.61227100	-0.33018600
6	0.76173200	-0.37790400	-0.19744700
7	-2.65065400	0.02514600	0.17079800
6	-1.39562400	-0.30434200	0.01872900
7	-0.29169300	0.48405600	0.03960300
6	-0.29984700	1.95139000	0.38291300
6	0.84008100	2.30873500	1.33458000
1	0.64171300	3.31218700	1.72485100
1	1.81108900	2.33510800	0.83492300

1	0.88318600	1.61511000	2.18119800
6	-0.34235100	2.78652100	-0.89409500
1	-1.23305800	2.07970600	0.93625700
1	0.58013900	2.66660200	-1.46888300
1	-0.45150200	3.84128400	-0.61955600
1	-1.19486500	2.50254100	-1.52047900
6	-3.79779500	-0.92725600	0.15463900
6	-4.73078000	-0.56449800	1.30814200
1	-4.21016100	-0.62523700	2.27007400
1	-5.57524700	-1.26164000	1.32285600
1	-5.12655400	0.45121000	1.18295300
6	-4.48278800	-0.86850000	-1.21096100
1	-3.37974800	-1.92280900	0.32173900
1	-5.32050200	-1.57456600	-1.22794300
1	-3.78814200	-1.13473400	-2.01523700
1	-4.87440800	0.13800100	-1.40187900
6	2.19166900	0.02819000	-0.45542700
8	3.03235000	-0.79624800	0.13839800
8	2.45908700	0.97370500	-1.16737100
6	4.46914700	-0.56491500	-0.10344900
6	5.22744100	-1.60148600	0.69228000
1	4.69607600	0.45649700	0.21417800
1	4.64030600	-0.65424800	-1.18002900
1	5.02604800	-1.50191500	1.76455100
1	6.30098800	-1.45308100	0.52716900
1	4.96553000	-2.61564800	0.37139200
1	-2.88238600	1.01574400	0.21537400

Table S17: total electronic energy, thermal correction to Gibbs free energy and atomic coordinates of **TS1-M1(E)**

Total electronic energy (Hartree): -913.983961220

Thermal correction to Gibbs Free Energy (Hartree): 0.271490

Atomic coordinates:

8	-1.36294600	-1.49972800	-0.17899400
7	-0.09420800	-1.66456400	-0.89030000
6	0.77136900	-0.97369000	-0.14004100

7	-2.73896200	0.23611200	0.37972600
6	-1.49164500	-0.16928600	0.12693500
7	-0.38464700	0.51161300	0.17746000
6	-0.24644900	1.94066100	0.48630400
6	1.18054800	2.22266100	0.96214400
1	1.26121500	3.27172000	1.26684500
1	1.90336200	2.05020600	0.15632300
1	1.44995300	1.59419400	1.81749800
6	-0.61208400	2.79421600	-0.73501700
1	-0.93127900	2.17624300	1.31497200
1	0.06879200	2.57621400	-1.56652100
1	-0.52861600	3.85880800	-0.48541500
1	-1.63820000	2.60029000	-1.06764200
6	-3.96902200	-0.51369700	0.03493300
6	-5.09178800	-0.02469300	0.94891900
1	-4.83802500	-0.17903300	2.00403300
1	-6.01380800	-0.57359100	0.72919400
1	-5.28643600	1.04420800	0.78904000
6	-4.30808400	-0.33982200	-1.44840200
1	-3.76928800	-1.56766800	0.24387600
1	-5.20811000	-0.91417800	-1.69891200
1	-3.48748300	-0.69310400	-2.08310000
1	-4.49617600	0.71666200	-1.67778200
6	2.05133300	-0.58261700	-0.88219700
8	3.08243700	-0.49378700	-0.04690400
8	2.07752400	-0.35933800	-2.07326700
6	4.35613300	-0.03341300	-0.60344800
6	5.32976400	0.08831600	0.54737500
1	4.17803400	0.92498900	-1.10053600
1	4.68192400	-0.76421600	-1.34964300
1	4.97400400	0.81380700	1.28743200
1	6.29615500	0.43310800	0.16128400
1	5.48005500	-0.87819700	1.04120400
6	0.94722800	-1.27839800	1.29212300
1	-2.86148400	1.22070800	0.59536000
7	1.05859700	-1.60741500	2.40140500

Table S18: total electronic energy, thermal correction to Gibbs free energy and atomic coordinates of **TS2-M1(E)**

Total electronic energy (Hartree): -913.992117802

Thermal correction to Gibbs Free Energy (Hartree): 0.271970

Atomic coordinates:

8	-1.10291900	-1.54652000	-0.28634900
7	0.36853000	-1.54675200	-0.45534200
6	0.73448900	-0.35660500	0.06930600
7	-2.74934300	0.00986400	0.06054900
6	-1.47240500	-0.30707900	-0.03189800
7	-0.41071200	0.50587600	0.11630200
6	-0.53055700	1.95847000	0.46589000
6	0.64481700	2.48035200	1.29145000
1	0.44196400	3.53337000	1.51466100
1	1.59123600	2.43344300	0.74711600
1	0.74839800	1.94894900	2.24002700
6	-0.76068900	2.77611600	-0.80819200
1	-1.41776800	2.02368200	1.10567000
1	0.13001800	2.75395500	-1.44197400
1	-0.97868900	3.81465800	-0.53435500
1	-1.61057700	2.38970100	-1.38219700
6	-3.87366900	-0.92513400	-0.19167100
6	-4.98789000	-0.60730200	0.80405700
1	-4.63525400	-0.71144800	1.83628300
1	-5.82539800	-1.29610700	0.65037400
1	-5.35712200	0.41649200	0.66027000
6	-4.33202000	-0.81189800	-1.64731700
1	-3.49347200	-1.93177000	-0.00020400
1	-5.15097600	-1.51625500	-1.83364500
1	-3.51363000	-1.04297200	-2.33847000
1	-4.69255600	0.20241900	-1.85862700
6	2.01648900	0.15366900	-0.58069100
8	3.05430900	-0.59535400	-0.24314500
8	2.02523400	1.08303800	-1.36354200
6	4.33323700	-0.29131000	-0.89209700

6	5.35031500	-1.27612900	-0.36035700
1	4.59383300	0.74400000	-0.65343100
1	4.19284100	-0.38214700	-1.97334700
1	5.46523400	-1.17693200	0.72476800
1	6.31996600	-1.07481100	-0.83081400
1	5.06054900	-2.30672400	-0.59399500
6	1.20230500	-0.56187000	1.90539600
1	-2.99161300	0.98530500	0.20630100
7	1.27202400	-1.07238800	2.95800700

The total electronic energies, thermal corrections to Gibbs free energy and atomic coordinates of the reactant, intermediates, and transition state structures in Mechanism **1** (Z) (Table S18-S22)

Table S19: total electronic energy, thermal correction to Gibbs free energy and atomic coordinates of **INT1-M1(Z)**

Total electronic energy (Hartree): -913.999075921

Thermal correction to Gibbs Free Energy (Hartree): 0.273637

Atomic coordinates:

8	-1.05914500	-1.00180900	1.19765300
7	0.29837000	-0.60198800	1.75143800
6	0.79240600	0.23144400	0.66006600
7	-2.65796300	-0.07275900	-0.14302200
6	-1.41418100	-0.10511700	0.31398600
7	-0.41565200	0.72183600	-0.03111300
6	-0.51785400	1.84281500	-1.00511000
6	-0.97006200	3.11933900	-0.28970500
1	-1.13328100	3.91473500	-1.02594500
1	-0.20960400	3.45775800	0.42260900
1	-1.90771700	2.95177500	0.25186500
6	0.78154500	2.03547600	-1.78893400
1	-1.28371300	1.54372100	-1.72803800
1	1.61271700	2.34308800	-1.14545600
1	0.62210000	2.83511400	-2.51973900
1	1.06374800	1.13013100	-2.33328300
6	-3.71426300	-1.03197900	0.25566300
6	-5.06284200	-0.32249300	0.14925700
1	-5.09221000	0.56766300	0.78788400
1	-5.86267200	-1.00201000	0.46234200
1	-5.26142300	-0.01797900	-0.88673900
6	-3.64591400	-2.29548400	-0.60629400
1	-3.52111300	-1.28968500	1.30070500
1	-4.41660700	-3.00671800	-0.28672200
1	-2.66890800	-2.78296900	-0.51510800
1	-3.81728700	-2.05002200	-1.66181400
6	1.65148100	-0.62646300	-0.31896200
8	2.93423800	-0.64532900	0.04393000

8	1.15659700	-1.29469400	-1.20797000
6	3.81446000	-1.58364800	-0.65158100
6	5.20185100	-1.41491400	-0.07223400
1	3.78197600	-1.35243300	-1.72059800
1	3.42168800	-2.59342400	-0.49662100
1	5.57096600	-0.39436900	-0.22502100
1	5.88689200	-2.10865600	-0.57391400
1	5.21008200	-1.63714800	1.00085200
6	1.57451300	1.32101700	1.29284800
1	-2.86472600	0.53540100	-0.92935100
7	2.15649300	2.16301400	1.83972300

Table S20: total electronic energy, thermal correction to Gibbs free energy and atomic coordinates of **INT2-M1(Z)**

Total electronic energy (Hartree): -821.045033396

Thermal correction to Gibbs Free Energy (Hartree): 0.270647

Atomic coordinates:

8	1.41373600	-1.60212500	-0.43125900
7	0.06908000	-2.08001600	-0.36840900
6	-0.61373100	-1.05723000	0.03585400
7	2.52778000	0.37880300	-0.04358600
6	1.42534700	-0.32107100	-0.05616800
7	0.16347300	0.06116700	0.26670100
6	-0.19867000	1.40236700	0.84900800
6	-0.59475500	2.36939800	-0.26406400
1	-0.75194800	3.36075800	0.17442500
1	-1.52105200	2.05216200	-0.75051900
1	0.19630400	2.44644700	-1.01756200
6	-1.23460000	1.26767000	1.96354800
1	0.73131300	1.74025700	1.31260500
1	-2.24337200	1.08980700	1.58342600
1	-1.25546000	2.21594700	2.51001000
1	-0.96346100	0.47470700	2.66849700
6	3.88967100	-0.14288100	-0.35181000
6	4.52549900	0.77216300	-1.39621000
1	3.92511900	0.80107100	-2.31204400

1	5.52468500	0.39877900	-1.64466100
1	4.62761100	1.79305200	-1.00732700
6	4.69510000	-0.22583500	0.94484300
1	3.75598300	-1.14236000	-0.77156800
1	5.69412900	-0.61978400	0.72755900
1	4.20843800	-0.88925300	1.66830600
1	4.80549800	0.76837600	1.39469900
6	-2.11437000	-1.17337500	0.16538400
8	-2.70373400	-0.18829000	-0.49045000
8	-2.64054100	-2.08378800	0.76588000
6	-4.17533500	-0.12614600	-0.43747200
6	-4.59104600	1.13842300	-1.15277200
1	-4.46795000	-0.12795700	0.61619100
1	-4.56020700	-1.02867400	-0.91980400
1	-4.18887000	2.02613300	-0.65311400
1	-5.68533200	1.20256800	-1.14335500
1	-4.25486200	1.13120900	-2.19531500
1	2.46675600	1.35981200	0.22239800

Table S21: total electronic energy, thermal correction to Gibbs free energy and atomic coordinates of **TS1-M1(Z)**

Total electronic energy (Hartree): -913.981990431

Thermal correction to Gibbs Free Energy (Hartree): 0.272386

Atomic coordinates:

8	-1.31434800	-0.70408400	1.27240600
7	-0.24343000	-0.23075800	2.15130200
6	0.79286400	-0.04831800	1.31191400
7	-2.49280100	0.15893000	-0.47710300
6	-1.32155300	0.07454800	0.15921800
7	-0.18698300	0.64343800	-0.15046600
6	-0.00696000	1.76109200	-1.08731000
6	-0.25076700	3.09603700	-0.37324900
1	-0.15276400	3.92608500	-1.08318000
1	0.48181500	3.23868600	0.42980500
1	-1.25634800	3.12996100	0.06161300
6	1.38092300	1.69493300	-1.72596800

1	-0.74973600	1.64400400	-1.88950300
1	2.16527600	1.80950000	-0.97121700
1	1.48296800	2.50526300	-2.45662700
1	1.53366100	0.74080400	-2.24093000
6	-3.74038800	-0.52098100	-0.06535800
6	-4.91942400	0.27490100	-0.62396500
1	-4.90887500	1.30737000	-0.25615600
1	-5.86112200	-0.19355900	-0.31805400
1	-4.89106600	0.29449900	-1.72161700
6	-3.74953900	-1.97908300	-0.53516400
1	-3.76779900	-0.49004300	1.02738200
1	-4.67059400	-2.47484100	-0.20568500
1	-2.89614400	-2.52937100	-0.12395600
1	-3.70271300	-2.02933800	-1.63047100
6	1.42921300	-1.15997600	0.45118400
8	2.57204500	-0.73732800	-0.08737800
8	0.95133500	-2.26693900	0.31649700
6	3.25395800	-1.63983500	-1.01341600
6	4.48094600	-0.91590400	-1.52288100
1	2.55491900	-1.88627700	-1.81868400
1	3.50782900	-2.55686500	-0.47333200
1	4.20484900	0.00367300	-2.05040700
1	5.01708600	-1.56878200	-2.22175500
1	5.15793400	-0.66185200	-0.69943800
6	1.75391900	0.89221600	1.87815200
1	-2.47551000	0.54363700	-1.41709000
7	2.48534700	1.67645900	2.32314500

Table S22: total electronic energy, thermal correction to Gibbs free energy and atomic coordinates of **TS2-M1(Z)**

Total electronic energy (Hartree): -913.991125207

Thermal correction to Gibbs Free Energy (Hartree): 0.272702

Atomic coordinates:

8	-1.13913000	-1.44180500	0.63940900
7	0.33533800	-1.54552700	0.59015700
6	0.72460100	-0.32196500	0.18013300

7	-2.74813600	-0.03487500	-0.18615800
6	-1.48031800	-0.34153200	0.00168800
7	-0.39861100	0.36889500	-0.38056000
6	-0.50335300	1.78588000	-0.86644200
6	-0.97001600	2.73366100	0.24795700
1	-1.24096100	3.69259600	-0.20774300
1	-0.17442400	2.91111600	0.97588800
1	-1.84849600	2.35372600	0.77960300
6	0.78824000	2.27665700	-1.51375100
1	-1.26891000	1.74499100	-1.64955900
1	1.61662900	2.30578400	-0.79722000
1	0.61603700	3.30151100	-1.85895400
1	1.07419500	1.66503000	-2.37105700
6	-3.89734600	-0.88814000	0.20518600
6	-4.96348000	0.00601800	0.83683300
1	-4.56881700	0.52799900	1.71563900
1	-5.81840900	-0.60420900	1.14753100
1	-5.32080700	0.75234000	0.11554300
6	-4.41351200	-1.65313500	-1.01528500
1	-3.52467300	-1.59223700	0.95280500
1	-5.25320200	-2.29393700	-0.72254900
1	-3.62864100	-2.28532800	-1.44555500
1	-4.76386200	-0.95568100	-1.78635100
6	2.01377600	-0.44434800	-0.64057100
8	3.08455900	-0.54750500	0.13017200
8	1.99027800	-0.53812400	-1.85112800
6	4.36131000	-0.81030800	-0.53996400
6	5.41818300	-0.91954000	0.53634200
1	4.55543200	0.01699600	-1.22908800
1	4.25699400	-1.73464000	-1.11593600
1	5.49394400	0.01069700	1.11035800
1	6.38813400	-1.11444400	0.06379300
1	5.19689500	-1.74366000	1.22377500
6	1.23106400	0.78490300	1.68074500
1	-2.96707500	0.80691400	-0.71073900
7	1.35990300	1.09793400	2.80298400

Table S23: total electronic energy, thermal correction to Gibbs free energy and atomic coordinates of **Z-6-M1**

Total electronic energy (Hartree): -914.004160256

Thermal correction to Gibbs Free Energy (Hartree): 0.272072

Atomic coordinates:

8	1.05668700	-1.32800300	-0.80218800
7	-0.10919900	-1.71866700	-1.44865600
6	-1.21455500	-1.48464400	-0.83211500
7	2.52110800	0.22049700	-0.07871400
6	1.25108200	0.04277900	-0.53258300
7	0.32277800	0.87853800	-0.77087800
6	0.52703300	2.30960000	-0.51636000
6	1.29580000	2.95864700	-1.67750600
1	1.45045300	4.02749600	-1.48466600
1	0.72889600	2.85467100	-2.61153100
1	2.27790400	2.49207400	-1.81866900
6	-0.83701400	2.97378900	-0.31740400
1	1.10787500	2.45672200	0.41056700
1	-1.46590100	2.83102000	-1.20550300
1	-0.71807800	4.05063000	-0.14559700
1	-1.35948900	2.54505300	0.54379300
6	3.38870800	-0.83603100	0.48620600
6	4.81742900	-0.29419000	0.53428400
1	5.16286900	-0.00073500	-0.46391200
1	5.49535600	-1.06110800	0.92490700
1	4.88015700	0.58088700	1.19533900
6	2.89683100	-1.28820400	1.86587100
1	3.35479500	-1.68260100	-0.20528400
1	3.53361100	-2.09419600	2.25151100
1	1.86600700	-1.65313200	1.81186500
1	2.93159700	-0.45160800	2.57619200
6	-1.42399300	-0.90000100	0.55708800
8	-2.52187800	-0.15873100	0.58110500
8	-0.70714200	-1.17169500	1.49685300
6	-2.88164300	0.45647300	1.86415100

6	-4.09168400	1.32840100	1.61833300
1	-2.01877900	1.02853800	2.21591500
1	-3.08557800	-0.35078000	2.57395400
1	-3.86817300	2.11552000	0.89008900
1	-4.38401100	1.80283200	2.56242800
1	-4.93759600	0.73533800	1.25329400
6	-2.38859800	-1.92185500	-1.54627000
1	2.70716700	1.15337000	0.27604600
7	-3.35137200	-2.26339200	-2.09876300

The total electronic energies, thermal corrections to Gibbs free energy and atomic coordinates of the reactant, intermediates, and transition state structures in Mechanism **2** (Table S23-S30)

Table S24: total electronic energy, thermal correction to Gibbs free energy and atomic coordinates of **11**

Total electronic energy (Hartree): -820.560634094

Thermal correction to Gibbs Free Energy (Hartree): 0.255479

Atomic coordinates:

7	-0.99893500	-1.73607300	-0.47368200
6	-1.75831300	-0.70655300	-0.36145700
8	0.38499700	-1.47704200	-0.52846900
6	-1.26433600	0.71524300	-0.32521400
8	-2.04928700	1.65222600	-0.42610000
7	0.09812000	0.85124400	-0.19659900
6	0.92807400	-0.27642100	-0.09191700
7	2.12845200	-0.21150900	0.29294100
6	2.98050100	-1.40830500	0.33931600
6	4.01795500	-1.21054600	1.44674100
6	3.65010700	-1.61894200	-1.02631000
1	2.38004200	-2.29818600	0.57317400
1	3.53052300	-1.05886600	2.41755300
1	4.66773600	-2.09094400	1.52229800
1	4.64437500	-0.33405400	1.23625300
1	2.90332300	-1.78032600	-1.81239400
1	4.25398700	-0.74362900	-1.29702400
1	4.30882600	-2.49540400	-0.99319000
6	0.66409400	2.23921100	-0.10674100
6	1.74907000	2.48216900	-1.15706100
6	1.09754200	2.57781500	1.32210600
1	-0.19039100	2.86711100	-0.35813700
1	1.39948500	2.18860100	-2.15392000
1	1.97196700	3.55531200	-1.17702500
1	2.67061000	1.94113700	-0.93122300
1	0.28672200	2.37581100	2.03200200
1	1.98060800	2.00834900	1.62157700
1	1.33582200	3.64689000	1.37218400
8	-3.07824500	-0.93260800	-0.40380000

6	-3.84976100	-0.55947200	0.78898900
6	-5.31312500	-0.73136700	0.45049800
1	-3.61635700	0.47424600	1.05580800
1	-3.53822600	-1.22645400	1.60117300
1	-5.91610100	-0.48264900	1.33194300
1	-5.60510900	-0.06440200	-0.36858400
1	-5.53186700	-1.76578400	0.16216400

Table S25: total electronic energy, thermal correction to Gibbs free energy and atomic coordinates of **INT1-M2**

Total electronic energy (Hartree): -913.993254028

Thermal correction to Gibbs Free Energy (Hartree): 0.273092

Atomic coordinates:

7	0.57523700	-2.15509200	0.39108100
6	1.57350800	-1.49132100	-0.06981000
6	2.70783100	-2.27853000	-0.46844800
7	3.64496300	-2.88589700	-0.78786300
8	-0.51865000	-1.34385500	0.78131400
6	1.65653600	0.04542200	-0.24749800
8	2.47095000	0.47900500	-1.10681900
7	0.08657700	0.34329300	-0.68341400
6	-0.83933900	-0.25280700	0.02548300
7	-2.12691000	0.06550300	0.11065200
1	-2.39311400	0.99360800	-0.19982700
6	-3.20834600	-0.77394400	0.67942900
6	-4.47785300	-0.52574000	-0.13509500
6	-3.39637800	-0.46466800	2.16667100
1	-2.89764100	-1.81447400	0.55721700
1	-4.32622400	-0.77920900	-1.19026500
1	-5.29306500	-1.14324300	0.25717500
1	-4.78496900	0.52624100	-0.06765100
1	-2.47419500	-0.65332000	2.72718400
1	-3.68226700	0.58523500	2.30753400
1	-4.18928200	-1.09766100	2.58214200
6	-0.14835100	1.18791000	-1.87811600
6	-0.45378600	2.64990800	-1.53298800

6	-1.17513600	0.57553400	-2.83720900
1	0.83248800	1.16146100	-2.36196200
1	0.34277400	3.08592600	-0.92314500
1	-0.52234900	3.22558200	-2.46372900
1	-1.40348400	2.77032300	-0.99843300
1	-0.95227800	-0.48097700	-3.02597600
1	-2.20341200	0.65443700	-2.46958900
1	-1.12234800	1.11195800	-3.79132300
8	1.74006500	0.48608200	1.11130100
6	1.96236600	1.89388100	1.29808500
6	2.33067400	2.11760800	2.75365700
1	1.04465100	2.43994400	1.04241000
1	2.76229000	2.22956500	0.62886400
1	2.49119000	3.18765000	2.93567000
1	1.53061600	1.77159300	3.41928800
1	3.25227400	1.58133800	3.00989500

Table S26: total electronic energy, thermal correction to Gibbs free energy and atomic coordinates of **INT2-M2**

Total electronic energy (Hartree): -913.992517597

Thermal correction to Gibbs Free Energy (Hartree): 0.275371

Atomic coordinates:

7	1.00603100	-0.68707000	-1.87022200
6	1.77054700	-0.28848300	-0.74216500
6	3.15296000	-0.13241300	-1.27192100
7	4.24664600	-0.08039900	-1.65467500
8	-0.38874300	-0.82637900	-1.35995500
6	1.25590100	1.07444000	-0.25373300
8	1.95465100	2.06115900	-0.08507900
7	-0.13608400	1.08644800	-0.05930300
6	-0.87036800	0.01129100	-0.48624700
7	-2.10682200	-0.21665100	-0.06784500
1	-2.44573500	0.29455400	0.73853600
6	-2.95735100	-1.33314700	-0.54480800
6	-4.41720300	-0.92828000	-0.34963800
6	-2.60020500	-2.62449000	0.19678800

1	-2.75242600	-1.45215100	-1.61214300
1	-4.65387500	-0.01613500	-0.90887600
1	-5.07034300	-1.73247600	-0.70427300
1	-4.63487400	-0.75563600	0.71252200
1	-1.54599500	-2.88359200	0.05272000
1	-2.79161000	-2.51429400	1.27130400
1	-3.21366100	-3.45034000	-0.18173000
6	-0.74446300	2.32252700	0.55607800
6	-1.12265700	2.10895900	2.02450300
6	-1.86113600	2.90527600	-0.31063100
1	0.08944100	3.02309200	0.53127500
1	-0.29538100	1.64836000	2.57509700
1	-1.33251000	3.08685500	2.47196100
1	-2.01772800	1.49275100	2.16354500
1	-1.52639300	3.01970000	-1.34756800
1	-2.77535600	2.30484500	-0.30151000
1	-2.11020800	3.89766900	0.08106000
8	1.75541600	-1.26293300	0.31618500
6	2.35503200	-0.86601900	1.57190500
6	2.27923800	-2.05461700	2.51011200
1	1.81112800	-0.00937200	1.99327900
1	3.39814500	-0.56278100	1.40438600
1	2.71930800	-1.78897300	3.47891300
1	1.23728300	-2.35376000	2.67356500
1	2.83002600	-2.90965000	2.10157600

Table S27: total electronic energy, thermal correction to Gibbs free energy and atomic coordinates of **INT3-M2**

Total electronic energy (Hartree): -821.011933923

Thermal correction to Gibbs Free Energy (Hartree): 0.270288

Atomic coordinates:

7	-1.04615000	-1.75034200	-0.13248500
6	-1.79980700	-0.70916300	-0.25741100
8	0.34757700	-1.49193600	-0.13409600
6	-1.28128800	0.69794200	-0.33924900
8	-2.00854700	1.63565700	-0.59418900

7	0.08961900	0.83947600	-0.07508900
6	0.87008400	-0.27199400	-0.00816900
7	2.16734100	-0.25483800	0.16061600
1	2.64518500	0.63901900	0.15063000
6	3.05450700	-1.45265900	0.23815600
6	4.20632000	-1.10941400	1.17929900
6	3.52163800	-1.83207300	-1.16796500
1	2.45821800	-2.25970200	0.66846400
1	3.83678000	-0.84322100	2.17565600
1	4.86435500	-1.97938700	1.27220900
1	4.79772100	-0.27321100	0.78539100
1	2.67363700	-2.06748400	-1.82026400
1	4.09480100	-1.01215300	-1.61667300
1	4.16685800	-2.71555900	-1.10869300
6	0.62962500	2.25544400	0.04027800
6	1.45457400	2.64238200	-1.18845100
6	1.30921300	2.50244400	1.38959100
1	-0.28519700	2.84482000	0.02469500
1	0.90164500	2.43135900	-2.10977800
1	1.64350800	3.72009000	-1.13815400
1	2.42691300	2.14165600	-1.24155800
1	0.70455400	2.10401200	2.21089900
1	2.32098500	2.09287000	1.46393800
1	1.39058800	3.58693200	1.51831700
8	-3.09074100	-0.95773500	-0.39191400
6	-4.09267200	-0.14519100	0.34095200
6	-5.27050100	-1.05230100	0.61179000
1	-4.35590600	0.69983100	-0.29581300
1	-3.63429300	0.21611100	1.26553300
1	-6.04692600	-0.47346200	1.12661800
1	-5.69200000	-1.43743400	-0.32343400
1	-4.98356300	-1.89555600	1.24938200

Table S28: total electronic energy, thermal correction to Gibbs free energy and atomic coordinates of **TS1-M2**

Total electronic energy (Hartree): -913.993172073

Thermal correction to Gibbs Free Energy (Hartree): 0.272885

Atomic coordinates:

7	-0.55702800	-2.09279300	-0.53353100
6	-1.58594400	-1.49236300	-0.04917800
6	-2.68037000	-2.35980200	0.29942600
7	-3.58482500	-3.03083500	0.58363100
8	0.52090000	-1.25311500	-0.87037400
6	-1.78089400	0.01321200	0.18892000
8	-2.55439400	0.38414800	1.09300000
7	-0.06761900	0.32931300	0.70277500
6	0.85568900	-0.22404400	-0.01849300
7	2.16039400	0.05630000	-0.07318700
1	2.45250000	0.94292900	0.32119900
6	3.22150200	-0.76850200	-0.69470800
6	4.49388200	-0.60807000	0.13780800
6	3.42818600	-0.36704900	-2.15763000
1	2.88631100	-1.80761000	-0.64479300
1	4.33085400	-0.92599800	1.17382900
1	5.29598200	-1.21865100	-0.29087700
1	4.82802600	0.43789300	0.14125500
1	2.50460700	-0.49261000	-2.73336700
1	3.74271900	0.68195100	-2.22701700
1	4.20642000	-0.99176300	-2.61208900
6	0.13889300	1.15063700	1.90615800
6	0.67978800	2.55687900	1.60966100
6	0.97277900	0.41524800	2.96286900
1	-0.88100900	1.26155300	2.29017100
1	0.11324000	3.04227600	0.80859900
1	0.58881000	3.17116800	2.51312200
1	1.73967000	2.55116700	1.32718100
1	0.54507000	-0.57145700	3.17480400
1	2.01374100	0.28296800	2.64744700
1	0.97429000	0.99709200	3.89193300
8	-1.81171200	0.56966800	-1.09170000
6	-2.20198400	1.95753300	-1.15328700
6	-2.08846900	2.39955000	-2.59946700

1	-1.54050300	2.54604400	-0.50712800
1	-3.22660200	2.06087500	-0.77951700
1	-2.38836700	3.45095300	-2.68780800
1	-1.05747400	2.30243600	-2.95976800
1	-2.74141800	1.79990200	-3.24486300

Table S29: total electronic energy, thermal correction to Gibbs free energy and atomic coordinates of **TS2-M2**

Total electronic energy (Hartree): -913.971280678

Thermal correction to Gibbs Free Energy (Hartree): 0.273275

Atomic coordinates:

7	0.72724900	-1.66049100	-1.22375200
6	1.52551600	-0.65367300	-0.98298000
6	2.88795700	-0.87558700	-1.39286900
7	3.98477000	-1.05427300	-1.72743900
8	-0.63428700	-1.38218000	-0.84040400
6	1.12563700	0.73982100	-0.69885200
8	1.86959100	1.69763100	-0.85727000
7	-0.26431600	0.89756500	-0.42538400
6	-1.05008500	-0.19994400	-0.39522600
7	-2.29800500	-0.20450500	0.02935000
1	-2.66510400	0.63118600	0.46732500
6	-3.20416900	-1.38444400	0.03012700
6	-4.63616300	-0.86786100	-0.09139700
6	-2.97897700	-2.21591100	1.29467400
1	-2.95625600	-1.97644800	-0.85373700
1	-4.76938100	-0.28322800	-1.00855700
1	-5.32686500	-1.71702900	-0.11678200
1	-4.89969700	-0.23918100	0.76892400
1	-1.94302100	-2.56513300	1.36084100
1	-3.20832500	-1.62397500	2.18895200
1	-3.63796500	-3.09157200	1.27947100
6	-0.71669000	2.27996200	-0.03233800
6	-0.86842200	2.41135300	1.48570700
6	-1.91823500	2.76833300	-0.84748200
1	0.13775400	2.88495900	-0.33188300

1	0.03786900	2.06449500	1.99263200
1	-1.01936500	3.46935500	1.72761700
1	-1.72376500	1.85566700	1.88519900
1	-1.78699200	2.54011800	-1.91076500
1	-2.88102300	2.36544400	-0.51839800
1	-1.97246600	3.85673200	-0.73601200
8	1.72000800	-0.40388900	1.06125500
6	2.84021000	0.19168400	1.64907000
6	3.91852500	-0.82249000	2.04358000
1	2.52262800	0.75236900	2.55031300
1	3.28052800	0.94950700	0.97064800
1	4.77405600	-0.32063800	2.51813300
1	3.51314900	-1.55588700	2.75279500
1	4.28325100	-1.36477200	1.16311600

Table S30: total electronic energy, thermal correction to Gibbs free energy and atomic coordinates of **TS3-M2**

Total electronic energy (Hartree): -913.971109028

Thermal correction to Gibbs Free Energy (Hartree): 0.272258

Atomic coordinates:

7	-0.92103200	-1.39583200	-1.06772900
6	-1.66851400	-0.50655100	-0.37277400
6	-1.65604300	-0.99609700	1.50823100
7	-1.35463600	-1.55132300	2.49479800
8	0.47257500	-1.30085600	-0.63738800
6	-1.10159800	0.89916000	-0.22891300
8	-1.80322400	1.89272000	-0.23159800
7	0.29631300	0.96813200	-0.13845700
6	1.01495500	-0.19211100	-0.19845200
7	2.28218700	-0.27637000	0.15772200
1	2.72664600	0.53336500	0.57236600
6	3.12935500	-1.48717400	-0.00179900
6	4.24916500	-1.40835000	1.03328100
6	3.65436900	-1.57882100	-1.43641000
1	2.49439100	-2.34997700	0.21618900
1	3.84465500	-1.34934500	2.05011100

1	4.87408300	-2.30422900	0.96031900
1	4.88563200	-0.53201300	0.85466600
1	2.83205600	-1.62111100	-2.15882200
1	4.28473900	-0.71291400	-1.67118400
1	4.25573400	-2.48830200	-1.54808500
6	0.91958700	2.33383900	0.03065500
6	1.97887200	2.61764300	-1.03585800
6	1.36762100	2.58150500	1.47333900
1	0.07646200	2.99388400	-0.16714100
1	1.59671200	2.37632500	-2.03372200
1	2.20375900	3.68933300	-1.00529700
1	2.91976700	2.08155300	-0.88247600
1	0.56518900	2.33229400	2.17591500
1	2.26415400	2.02077000	1.75958100
1	1.60200700	3.64627800	1.58090400
8	-2.95370300	-0.60669200	-0.77737600
6	-4.03157300	0.06015400	-0.05955700
6	-5.28193900	-0.77168100	-0.25827900
1	-4.14341500	1.06815500	-0.46445800
1	-3.76349600	0.13447000	0.99908900
1	-6.12531800	-0.27707400	0.23919400
1	-5.52247300	-0.87168600	-1.32319100
1	-5.16183800	-1.77250900	0.17181900

Table S31: total electronic energy, thermal correction to Gibbs free energy and atomic coordinates of **Z-6-M2**

Total electronic energy (Hartree): -914.000632092

Thermal correction to Gibbs Free Energy (Hartree): 0.272616

Atomic coordinates:

7	-1.13486200	-0.24925400	-1.96022700
6	-2.09105700	-0.11453700	-1.10714900
6	-3.37953800	-0.51800800	-1.62052100
7	-4.42378500	-0.83526300	-2.01688500
8	0.13733200	0.11172400	-1.54603000
6	-2.11387800	0.39913900	0.32362800
8	-2.89272100	-0.05622900	1.13841600

7	-0.01807500	-1.29766600	0.28830000
6	0.72016500	-0.58455000	-0.45501900
7	2.05450900	-0.33023200	-0.46098100
1	2.54452400	-0.58911700	0.38598500
6	2.77283900	0.61726100	-1.33808100
6	4.25309100	0.23263800	-1.32909700
6	2.55636200	2.06989300	-0.89849000
1	2.37829300	0.48319300	-2.34899800
1	4.39506500	-0.79828500	-1.67380900
1	4.81741000	0.90050700	-1.98924500
1	4.67318400	0.32213300	-0.31815000
1	1.49243900	2.32693100	-0.90408000
1	2.94642800	2.22546300	0.11533200
1	3.08065800	2.75340700	-1.57806200
6	0.44495400	-2.12514400	1.40916600
6	0.99753400	-1.29850900	2.58210400
6	1.41857500	-3.23103700	0.97118300
1	-0.46141200	-2.62279400	1.77527200
1	0.28967100	-0.51460700	2.87288200
1	1.16002500	-1.95188200	3.44804500
1	1.95600900	-0.82042400	2.34697000
1	0.99413300	-3.81230300	0.14374400
1	2.38461400	-2.82864300	0.64539700
1	1.60474100	-3.91398500	1.80913400
8	-1.30169600	1.43117400	0.48993200
6	-1.25083000	2.01867800	1.83253600
6	-0.07138600	2.96410000	1.86806000
1	-1.15100900	1.20592300	2.55580700
1	-2.20217500	2.53171200	2.00487300
1	-0.03054800	3.43988100	2.85507300
1	0.86826400	2.42731200	1.70339700
1	-0.16912200	3.74957900	1.11052000

The total electronic energies, thermal corrections to Gibbs free energy and atomic coordinates of the reactant, intermediates, and transition state structures in Mechanism **3** (Table S31-S34)

Table S32: total electronic energy, thermal correction to Gibbs free energy and atomic coordinates of **6-M3**

Total electronic energy (Hartree): -914.011818150

Thermal correction to Gibbs Free Energy (Hartree): 0.270736

Atomic coordinates:

8	-0.35742800	-0.95366300	-0.04461800
7	0.67487200	-0.08125100	-0.00368900
6	1.82578000	-0.64735100	0.14636700
7	-1.64463400	0.95118600	-0.61285700
6	-1.66037500	-0.35077100	-0.20400600
7	-2.57061500	-1.20027300	0.01868600
6	-3.98033300	-0.95455500	-0.29973800
6	-4.52120500	-2.18910700	-1.03096600
1	-5.57727300	-2.04434200	-1.29071400
1	-4.43850900	-3.08024100	-0.39567000
1	-3.96130800	-2.37219800	-1.95604800
6	-4.76890300	-0.69120800	0.99004500
1	-4.08884900	-0.09255200	-0.97260500
1	-4.69972200	-1.55827000	1.65925900
1	-5.82689200	-0.51759200	0.75869100
1	-4.38478500	0.18399400	1.52563200
6	-2.51847000	1.99813800	-0.01514900
6	-2.13616000	2.26415400	1.44398300
1	-2.21072200	1.35005700	2.04453000
1	-2.80651900	3.01442200	1.88079400
1	-1.10659900	2.63870800	1.50920400
6	-2.39699400	3.25148600	-0.88049700
1	-3.54830800	1.64366600	-0.05911000
1	-3.04119500	4.04337400	-0.48259200
1	-2.69596700	3.04625900	-1.91492400
1	-1.36397400	3.62439400	-0.88390800
6	3.02232900	0.26939000	0.18975300
8	4.13561400	-0.44391700	0.35206900

8	2.94400100	1.47669400	0.09461000
6	5.41237300	0.27885800	0.39244900
6	5.91575200	0.56244800	-1.00994600
1	6.07224600	-0.40235600	0.93218800
1	5.27326600	1.19144300	0.97712200
1	6.02928200	-0.36704900	-1.57881400
1	6.89704200	1.04754000	-0.94169000
1	5.23788400	1.23148500	-1.54978400
6	1.99417400	-2.06883700	0.26729900
1	-0.70375400	1.30631000	-0.76687200
7	2.11742700	-3.22049400	0.36232600

Table S33: total electronic energy, thermal correction to Gibbs free energy and atomic coordinates of **INT-M3**

Total electronic energy (Hartree): -913.953330300

Thermal correction to Gibbs Free Energy (Hartree): 0.277078

Atomic coordinates:

8	-0.66782300	-0.16796200	2.07838100
7	0.73439600	-0.59074300	1.65671700
6	0.87922700	0.27167100	0.55125700
7	-0.49804000	0.17157300	-0.19134900
6	-1.37624100	-0.34243000	0.95351900
7	-2.51265300	-0.85827000	0.88448800
6	-3.27481000	-1.10496500	-0.34496800
6	-3.76979400	-2.55418100	-0.29786700
1	-4.33737300	-2.78327400	-1.20768100
1	-4.42228400	-2.71070100	0.56994000
1	-2.92807700	-3.25343300	-0.23057200
6	-4.44227200	-0.11530700	-0.42749900
1	-2.64300600	-0.99633800	-1.23727500
1	-5.09112800	-0.21906600	0.45077600
1	-5.03777300	-0.31927700	-1.32498200
1	-4.08856700	0.91982500	-0.47493600
6	-1.08405600	1.34580200	-1.01939300
6	-1.62639600	2.47980400	-0.15691100
1	-2.27623400	2.11551800	0.64445200

1	-2.23497400	3.11577000	-0.80895100
1	-0.84016300	3.10031600	0.27553900
6	-0.06336300	1.78446300	-2.06470600
1	-1.91971300	0.87292200	-1.53423000
1	-0.54313500	2.52945800	-2.70826000
1	0.25342000	0.94616700	-2.69312700
1	0.82078400	2.25112700	-1.61925800
6	1.97326100	-0.32754900	-0.36749600
8	3.17950100	-0.03581600	0.07294500
8	1.68598000	-1.01836000	-1.32651600
6	4.33265900	-0.63702600	-0.62202100
6	4.58314000	-2.04395500	-0.11777400
1	5.15217200	0.03988300	-0.37688100
1	4.13796700	-0.60348200	-1.69643300
1	4.75505000	-2.04576200	0.96411900
1	5.47907000	-2.43999100	-0.61098000
1	3.74263400	-2.70685800	-0.34733900
6	1.17901100	1.68631600	0.93504300
1	-0.35857700	-0.60573900	-0.85815800
7	1.55293100	2.69375300	1.37852300

Table S34: total electronic energy, thermal correction to Gibbs free energy and atomic coordinates of **TS1-M3**

Total electronic energy (Hartree): -913.950711275

Thermal correction to Gibbs Free Energy (Hartree): 0.275579

Atomic coordinates:

8	-0.72356500	-0.39422000	2.00266500
7	0.60217000	-0.93031700	1.56394500
6	0.95807300	0.03295900	0.67852400
7	-0.57856200	0.11353500	-0.26224400
6	-1.44281600	-0.39996000	0.83852000
7	-2.62552500	-0.81619200	0.80048500
6	-3.43591700	-0.93666600	-0.41763400
6	-4.04335400	-2.34406300	-0.42022600
1	-4.64759500	-2.48814100	-1.32390400
1	-4.68637500	-2.48814700	0.45691300

1	-3.25840000	-3.10944400	-0.40413500
6	-4.52471500	0.14153400	-0.42329900
1	-2.82174000	-0.83383700	-1.32217300
1	-5.14410300	0.06257600	0.47869800
1	-5.17066500	0.01015200	-1.29931300
1	-4.09459800	1.14737000	-0.46082300
6	-1.07249100	1.33565100	-1.03676900
6	-1.55077700	2.46851900	-0.13271600
1	-2.23724100	2.11813100	0.64418400
1	-2.09850500	3.18143800	-0.75838400
1	-0.72522900	3.00332500	0.33964900
6	0.02109800	1.76047100	-2.01445100
1	-1.92375200	0.96828900	-1.61338300
1	-0.37051800	2.56581900	-2.64487900
1	0.31836800	0.93309300	-2.66848500
1	0.90848500	2.14141700	-1.49702600
6	2.06872500	-0.45075700	-0.27418900
8	3.24009100	0.08477900	0.02233400
8	1.82984200	-1.25270300	-1.15383000
6	4.41072400	-0.33773000	-0.76541900
6	4.98622200	-1.62719900	-0.21481000
1	5.10164600	0.49971000	-0.65713200
1	4.10394100	-0.42757300	-1.81012100
1	5.26459800	-1.51145100	0.83843400
1	5.88791200	-1.88234500	-0.78452700
1	4.27472500	-2.45413400	-0.30754800
6	1.15797800	1.40286200	1.19536100
1	-0.42072300	-0.64368900	-0.94014600
7	1.42653700	2.40881400	1.71030100

Table S35: total electronic energy, thermal correction to Gibbs free energy and atomic coordinates of **TS2-M3**

Total electronic energy (Hartree): -913.932903819

Thermal correction to Gibbs Free Energy (Hartree): 0.273674

Atomic coordinates:

8	0.68047300	-0.28430100	-2.06430500

7	-0.72602200	-0.42255100	-1.70634900
6	-0.84039300	0.11160200	-0.51355600
7	0.49528100	0.12189300	0.18371600
6	1.39878100	-0.40451800	-0.92182300
7	2.54567300	-0.88031300	-0.86368500
6	3.36411900	-1.08341600	0.33812500
6	3.88549500	-2.52310200	0.30111600
1	4.49779600	-2.71655300	1.18965200
1	4.50122000	-2.68737600	-0.59146600
1	3.05630700	-3.24001900	0.28992800
6	4.50977500	-0.06528700	0.33870900
1	2.76851600	-0.96230100	1.25301800
1	5.12456200	-0.18426000	-0.56155900
1	5.14393300	-0.22779700	1.21769800
1	4.13327000	0.96277300	0.36757600
6	1.11150100	1.36337600	0.93987300
6	1.58085000	2.46136100	-0.00094700
1	2.21247700	2.07366900	-0.80616000
1	2.19710900	3.14258300	0.59680800
1	0.75933300	3.03239700	-0.43284800
6	0.19182100	1.81019000	2.06643900
1	1.98668800	0.89590200	1.38899000
1	0.74619400	2.54841300	2.65695800
1	-0.06967100	0.97568300	2.72366800
1	-0.72260300	2.28188300	1.70207300
6	-1.99631300	-0.34163500	0.35471600
8	-3.13573000	-0.33288800	-0.30565400
8	-1.81190200	-0.68779000	1.50805300
6	-4.33625800	-0.81995400	0.39192900
6	-4.43137300	-2.33015200	0.30273600
1	-5.15176700	-0.32589600	-0.13822700
1	-4.30255400	-0.46290600	1.42382300
1	-4.44562000	-2.65921300	-0.74212800
1	-5.36445400	-2.65370300	0.77974200
1	-3.59567300	-2.81448000	0.81836900
6	-1.42401800	2.08483400	-0.65058000

1	0.42718300	-0.60408200	0.91534200
7	-2.00763000	2.96458700	-1.16174500

The total electronic energies, thermal corrections to Gibbs free energy and atomic coordinates of the reactant, intermediates, and transition state structures in Mechanism **4** (Table S35-S36)

Table S36: total electronic energy, thermal correction to Gibbs free energy and atomic coordinates of **6-M4**

Total electronic energy (Hartree): -914.001621422

Thermal correction to Gibbs Free Energy (Hartree): 0.271485

Atomic coordinates:

8	0.53158800	-0.13158400	-1.58230700
7	-0.79291100	-0.07403800	-1.19809300
6	-1.17439200	-0.66589100	-0.11772500
7	1.32385100	0.86897400	0.37660600
6	1.58441400	-0.02209200	-0.62041700
7	2.57584000	-0.77885100	-0.85124300
6	3.82799600	-0.59479300	-0.10344200
6	3.84234800	-1.52143900	1.12135600
1	4.79057200	-1.41748400	1.66287600
1	3.73341500	-2.56798000	0.81016300
1	3.02386300	-1.28365500	1.81085200
6	5.00063200	-0.89182300	-1.04171100
1	3.92169000	0.44666800	0.24438300
1	4.95321800	-1.92812300	-1.40041800
1	5.95426700	-0.74933300	-0.51879900
1	4.98300400	-0.22535600	-1.91267200
6	0.67838600	2.19159900	0.20988100
6	-0.14558100	2.49459300	1.46134600
1	-0.92512800	1.73785300	1.61018700
1	-0.62703900	3.47479000	1.36923500
1	0.49607300	2.51193800	2.35218200
6	1.73050600	3.26833600	-0.07561800
1	0.00472100	2.11978300	-0.64820400
1	1.24751500	4.24185400	-0.22357700
1	2.30291300	3.02563100	-0.97853400
1	2.42984400	3.35553000	0.76587800
6	-2.62790500	-0.54675800	0.28963200
8	-3.32174500	0.24189700	-0.51569900

8	-3.04483400	-1.12594400	1.27637000
6	-4.75369100	0.40582200	-0.23214100
6	-5.55385800	-0.74745500	-0.80617900
1	-4.99873400	1.35211800	-0.71714800
1	-4.88349500	0.50589100	0.84819300
1	-5.38992200	-0.83599800	-1.88592200
1	-6.62033100	-0.55850000	-0.63319300
1	-5.29024600	-1.69536300	-0.32594200
6	-0.37378500	-1.49521800	0.74670900
1	1.97753800	0.82517900	1.15264500
7	0.23404200	-2.22461200	1.41569400

Table S37: total electronic energy, thermal correction to Gibbs free energy and atomic coordinates of **TS-M4**

Total electronic energy (Hartree): -913.942095942

Thermal correction to Gibbs Free Energy (Hartree): 0.272417

Atomic coordinates:

8	-0.77606100	-0.74337100	1.94066500
7	0.64357200	-0.47160700	1.71193700
6	0.73878000	-0.32880000	0.40256100
7	-0.56366900	0.24124100	-0.12073300
6	-1.51241100	-0.42043800	0.85402200
7	-2.73623300	-0.63315100	0.74277200
6	-3.54454000	-0.32393700	-0.44424700
6	-4.13222000	-1.63665000	-0.97153200
1	-4.74954000	-1.43758600	-1.85547200
1	-4.75895500	-2.11221900	-0.20730900
1	-3.33689500	-2.33582600	-1.25477100
6	-4.63827200	0.67122000	-0.04393600
1	-2.93953100	0.12785700	-1.24337200
1	-5.27507400	0.24566900	0.74112600
1	-5.26445600	0.90291200	-0.91344100
1	-4.20625900	1.60707400	0.32858000
6	-0.78506300	1.80368800	-0.13746200
6	0.00203700	2.51539200	0.95272500
1	-0.19556800	2.10252700	1.94539700

1	-0.32658900	3.56037200	0.94739600
1	1.08026900	2.50850100	0.76870000
6	-0.54089900	2.33909400	-1.54187600
1	-1.84917200	1.88255500	0.08857000
1	-0.91153600	3.36961600	-1.56793200
1	-1.09745200	1.76301900	-2.29043200
1	0.51793000	2.34012300	-1.80454800
6	2.04026400	0.19848400	-0.18456800
8	3.04583700	0.02281400	0.65470700
8	2.09467700	0.66840700	-1.30316600
6	4.39276700	0.38438200	0.18692000
6	4.99777600	-0.75013500	-0.61629600
1	4.93967200	0.56808500	1.11299000
1	4.32324900	1.31199400	-0.38581600
1	5.03907300	-1.66962900	-0.02209100
1	6.02082000	-0.47581500	-0.90088500
1	4.42601200	-0.94224600	-1.53009800
6	0.68294500	-1.97200900	-0.53068100
1	-0.74958700	-0.11257900	-1.06700400
7	0.96932700	-3.08918900	-0.73126300

The total electronic energies, thermal corrections to Gibbs free energy and atomic coordinates of the most stable conformers of **6** (Table S37-S38)

Table S38: total electronic energy, thermal correction to Gibbs free energy and atomic coordinates of the most stable conformer of **E-6**

Total electronic energy (Hartree): -914.017949008

Thermal correction to Gibbs Free Energy (Hartree): 0.268721

Atomic coordinates:

8	-0.54416400	-0.51956800	-0.17310800
7	0.48047800	0.37156700	-0.11382500
6	1.63845600	-0.19728300	-0.08285300
7	-1.88897700	1.40569000	-0.26577800
6	-1.85374300	0.05423000	-0.20091100
7	-2.84961800	-0.73372500	-0.20011300
6	-2.68538400	-2.19521500	-0.19612000
6	-2.52303300	-2.70474900	1.24350200
1	-2.43508900	-3.79839200	1.25470800
1	-3.39369800	-2.42229000	1.84944000
1	-1.62567400	-2.28426400	1.71232400
6	-3.91253400	-2.81673100	-0.86828100
1	-1.79451500	-2.48970200	-0.76863600
1	-4.82594000	-2.55626500	-0.31747100
1	-3.82426900	-3.90985400	-0.89478300
1	-4.01918900	-2.45522100	-1.89843300
6	-3.14472500	2.16034300	-0.11632700
6	-3.58916300	2.21980300	1.34984400
1	-3.71624700	1.21260600	1.76187100
1	-4.54603900	2.74914400	1.43642900
1	-2.84381100	2.75337800	1.95379600
6	-2.94020200	3.55396000	-0.71120300
1	-3.90097300	1.62572600	-0.69984000
1	-3.86678500	4.13372900	-0.63433500
1	-2.65428000	3.49400700	-1.76789900
1	-2.15452200	4.09752100	-0.16931200
6	2.83720900	0.71594900	-0.01026100
8	3.95694600	-0.00195600	0.01384200

8	2.75707700	1.92636100	0.02020200
6	5.22264900	0.73667400	0.08395500
6	6.33784300	-0.28428800	0.09356900
1	5.20512500	1.34344100	0.99401200
1	5.27177400	1.39918700	-0.78511400
1	6.25828800	-0.94791200	0.96175500
1	7.29817000	0.24172700	0.14771500
1	6.32688900	-0.88976100	-0.81956800
6	1.81041800	-1.62370800	-0.11188900
1	-1.03076300	1.89795800	-0.04421100
7	1.93324800	-2.77886900	-0.13429700

Table S39: total electronic energy, thermal correction to Gibbs free energy and atomic coordinates of the most stable conformer of **Z-6**

Total electronic energy (Hartree): -914.012641204

Thermal correction to Gibbs Free Energy (Hartree): 0.268461

Atomic coordinates:

8	-0.34260200	0.15727200	0.04423200
7	0.26947100	-1.04702200	0.03952600
6	1.56246900	-1.00919500	-0.00735600
7	-2.30253900	-1.13233000	0.25221100
6	-1.77149000	0.10288800	0.09116600
7	-2.40953900	1.19902300	0.02290400
6	-1.72192500	2.49620900	-0.05831100
6	-2.61815800	3.46541200	-0.83372500
1	-2.13540100	4.44634800	-0.92469300
1	-3.57874200	3.59877400	-0.31878300
1	-2.82098800	3.08757100	-1.84343700
6	-1.42536800	3.02140100	1.35386900
1	-0.76898700	2.39973100	-0.59536100
1	-2.35515500	3.12245000	1.92874300
1	-0.94261100	4.00552800	1.30256100
1	-0.75745100	2.34042000	1.89442600
6	-3.74950400	-1.37880700	0.13388600
6	-4.19928400	-1.37627600	-1.33179700
1	-3.95039400	-0.42583000	-1.81667300

1	-5.28476200	-1.52049000	-1.39732500
1	-3.70950600	-2.18925000	-1.88336100
6	-4.06868800	-2.70229500	0.82986100
1	-4.24912100	-0.56222100	0.66450200
1	-5.14469500	-2.90298800	0.77895500
1	-3.76964500	-2.67587100	1.88429000
1	-3.54545300	-3.53544600	0.34146200
6	2.46092100	0.20596600	-0.06681000
8	3.73653200	-0.18223100	-0.05773600
8	2.06521600	1.35132900	-0.11775200
6	4.75310000	0.87302100	-0.11597100
6	6.10451100	0.19495100	-0.09532000
1	4.60378200	1.53023800	0.74584900
1	4.58865000	1.44650300	-1.03305600
1	6.24135200	-0.38413400	0.82471800
1	6.88637700	0.96214900	-0.14035000
1	6.22460100	-0.47167000	-0.95655800
6	2.16901900	-2.31414600	-0.00592800
1	-1.68980800	-1.91951500	0.07073400
7	2.65848700	-3.36796000	-0.00578200

The total electronic energies, thermal corrections to Gibbs free energy and atomic coordinates of the transition state structures of Oxyma ion isomerization (Table S39-S40)

Table S40: total electronic energy, thermal correction to Gibbs free energy and atomic coordinates of **ts** (inversion)

Total electronic energy (Hartree): -528.758831762

Thermal correction to Gibbs Free Energy (Hartree): 0.063760

Atomic coordinates:

8	3.47989400	-0.89283000	-0.00000100
7	2.31592700	-0.36688700	-0.00000100
6	1.15681600	0.11517500	-0.00000100
6	-0.11135500	-0.70026900	-0.00000000
8	-1.21329900	0.07928400	0.00000000
8	-0.14438000	-1.92168300	-0.00000000
6	-2.50306500	-0.59200000	0.00000100
6	-3.57651500	0.47641200	0.00000200
1	-2.56277100	-1.22985100	0.88817400
1	-2.56277300	-1.22985000	-0.88817400
1	-3.50103400	1.11074300	0.89077200
1	-4.56349100	-0.00192600	0.00000200
1	-3.50103500	1.11074500	-0.89076600
6	0.99451300	1.59453900	-0.00000100
7	1.10564700	2.76100500	-0.00000100

Table S41: total electronic energy, thermal correction to Gibbs free energy and atomic coordinates of **ts** (rotation)

Total electronic energy (Hartree): -528.783851755

Thermal correction to Gibbs Free Energy (Hartree): 0.064507

Atomic coordinates:

8	-2.97237900	-1.00612900	0.57253300
7	-2.36406400	-0.59696600	-0.39173900
6	-1.12034000	0.15778400	-0.05247400
6	0.08196500	-0.59784100	-0.10321600
8	1.20901000	0.17378600	0.01792700
8	0.13815900	-1.83034300	-0.23801200
6	2.47479000	-0.52430400	0.01085000
6	3.56724000	0.51491900	0.17344100

1	2.58051600	-1.07127000	-0.93283700
1	2.48998400	-1.25259400	0.82973700
1	3.54621300	1.23965200	-0.64910500
1	4.54640100	0.02037500	0.17215900
1	3.45631800	1.05756000	1.11972700
6	-1.23640700	1.53697500	-0.06149600
7	-1.38182700	2.70876100	-0.05996000

The total electronic energies, thermal corrections to Gibbs free energy and atomic coordinates of the conformers of **7** (Table S41-S48)

Table S42: total electronic energy, thermal correction to Gibbs free energy and atomic coordinates of conformer 1 of **7**

Total electronic energy (Hartree): -820.588800101

Thermal correction to Gibbs Free Energy (Hartree): 0.255531

Atomic coordinates:

8	0.79896600	-1.62133500	0.02988000
7	-0.61927600	-1.56262100	0.13062700
6	-0.89089000	-0.29138900	0.14115300
7	0.21097500	0.51322700	0.05433200
6	1.31450400	-0.33651500	-0.02749800
7	2.53669400	-0.01918700	-0.13536700
6	3.54923000	-1.08575900	-0.21535900
6	4.74764500	-0.55333000	-1.00409300
1	5.52255700	-1.32433500	-1.09604600
1	5.18438000	0.31727800	-0.49719000
1	4.44650200	-0.24602000	-2.01312800
6	3.96255100	-1.53035100	1.19519500
1	3.14424200	-1.95956100	-0.74787500
1	4.36899000	-0.68081900	1.75916300
1	4.73374500	-2.30902200	1.14119600
1	3.10729200	-1.93440800	1.74981600
6	0.25549600	1.99389300	-0.09942900
6	1.00873600	2.63160900	1.06942200
1	0.56527500	2.33460000	2.02660200
1	0.94346100	3.72209700	0.98237900
1	2.06447000	2.34571700	1.06489800
6	0.81957900	2.38074800	-1.46883900
1	-0.78646300	2.30798400	-0.05245000
1	0.72826400	3.46581800	-1.59264200
1	0.25618200	1.89419700	-2.27354800
1	1.87454100	2.10876300	-1.56083400
6	-2.29541700	0.19286900	0.36428000
8	-3.18060500	-0.62553700	-0.18714100
8	-2.54626200	1.19763800	1.00361900

6	-4.59778800	-0.31154900	0.03360800
6	-5.40713800	-1.36771600	-0.68471700
1	-4.78411600	0.69272400	-0.35769900
1	-4.77777500	-0.31163900	1.11277100
1	-5.20031600	-1.35939500	-1.76071400
1	-6.47306500	-1.15853100	-0.53631500
1	-5.19274600	-2.36655900	-0.28872900

Table S43: total electronic energy, thermal correction to Gibbs free energy and atomic coordinates of conformer 2 of **7**

Total electronic energy (Hartree): -820.588101923

Thermal correction to Gibbs Free Energy (Hartree): 0.255967

Atomic coordinates:

8	0.96290800	-1.62694200	-0.37958000
7	-0.45477400	-1.70836000	-0.46848200
6	-0.86041000	-0.48268300	-0.31581600
7	0.15182200	0.41828400	-0.13330100
6	1.34097900	-0.30961200	-0.17691800
7	2.52608200	0.12405100	-0.05843100
6	3.64491100	-0.83282800	-0.11064400
6	4.88925600	-0.09286700	-0.60597400
1	5.74511800	-0.77619900	-0.66858000
1	5.14836200	0.72418100	0.08028100
1	4.71890100	0.33713300	-1.60065000
6	3.87822200	-1.45084600	1.27573800
1	3.42088400	-1.64575600	-0.81775000
1	4.10338200	-0.66652200	2.00993700
1	4.72381900	-2.14950700	1.24709300
1	2.99310700	-1.99978600	1.61858500
6	0.04606200	1.86784500	0.19078900
6	0.60564800	2.15282200	1.58676700
1	0.11574400	1.52289100	2.33836200
1	0.40978700	3.20175100	1.83659200
1	1.68462400	1.98144700	1.63022300
6	0.69438000	2.71250300	-0.90780900
1	-1.02367700	2.07197000	0.19785300

1	0.51769100	3.77241500	-0.69235800
1	0.25619000	2.48138200	-1.88541100
1	1.77390700	2.54309300	-0.95487200
6	-2.31479300	-0.13033300	-0.45537600
8	-3.08666300	-1.11863600	-0.02255500
8	-2.68805700	0.92110800	-0.94104900
6	-4.54105600	-0.95036800	-0.14440900
6	-5.09247500	-0.18636400	1.04399800
1	-4.91251400	-1.97596200	-0.17732800
1	-4.75542200	-0.45410800	-1.09385600
1	-4.84873600	-0.69679000	1.98242600
1	-6.18417900	-0.13189400	0.95413000
1	-4.69874200	0.83458100	1.08021400

Table S44: total electronic energy, thermal correction to Gibbs free energy and atomic coordinates of conformer 3 of **7**

Total electronic energy (Hartree): -820.588211738

Thermal correction to Gibbs Free Energy (Hartree): 0.255066

Atomic coordinates:

8	-1.04169200	-1.58024700	-0.14180900
7	0.37705800	-1.59449400	-0.28067400
6	0.72572400	-0.34510100	-0.19374700
7	-0.32325300	0.50982600	-0.00015800
6	-1.47139200	-0.28174800	0.04501800
7	-2.66455500	0.10691000	0.22716600
6	-3.74668000	-0.89188400	0.26507000
6	-4.88726000	-0.33727900	1.12101700
1	-5.70938600	-1.06082100	1.18324400
1	-5.27710200	0.59226000	0.68553000
1	-4.54249300	-0.11989500	2.13942800
6	-4.22288600	-1.21345500	-1.15891800
1	-3.38731000	-1.82372300	0.72767400
1	-4.58685400	-0.30398200	-1.65419400
1	-5.04181700	-1.94324500	-1.13321800
1	-3.41008100	-1.63333800	-1.76330700
6	-0.36613300	1.97003500	0.28052900

6	0.67003000	2.37838300	1.32879600
1	0.59730800	1.74474700	2.22001800
1	0.47198100	3.41358500	1.62768100
1	1.69271300	2.33475900	0.94350900
6	-0.28819600	2.78181600	-1.01370400
1	-1.36052300	2.10181600	0.71435800
1	-0.45042300	3.84120000	-0.78323900
1	-1.06388100	2.46166800	-1.71866700
1	0.69011700	2.67196300	-1.48930500
6	2.15706500	0.05326800	-0.42269600
8	2.98889800	-0.79854500	0.16040300
8	2.46843300	1.01872800	-1.09258200
6	4.42378600	-0.57186300	-0.05531500
6	5.16597600	-1.64692600	0.70607500
1	4.66208000	0.43304200	0.30512800
1	4.61267900	-0.61805000	-1.13196500
1	4.95171800	-1.58985200	1.77913700
1	6.24317700	-1.50375000	0.56089100
1	4.89802300	-2.64503500	0.34212400

Table **S45**: total electronic energy, thermal correction to Gibbs free energy and atomic coordinates of conformer 4 of **7**

Total electronic energy (Hartree): -820.587577532

Thermal correction to Gibbs Free Energy (Hartree): 0.256026

Atomic coordinates:

8	1.04564200	-1.57630300	-0.16133300
7	-0.37924900	-1.61907500	-0.16290100
6	-0.74308100	-0.37364500	-0.08069400
7	0.30225600	0.50578400	-0.03211600
6	1.46509300	-0.26275700	-0.09568800
7	2.66213800	0.15554300	-0.10040500
6	3.76218600	-0.81927900	-0.19846200
6	4.95888700	-0.13356300	-0.86101000
1	5.79693000	-0.83409400	-0.96229600
1	5.29348900	0.72118800	-0.25845800
1	4.69516800	0.23429900	-1.86022000

6	4.12519700	-1.35179600	1.19529500
1	3.46008100	-1.67164500	-0.82579800
1	4.43153800	-0.52667000	1.85126000
1	4.95584400	-2.06554000	1.12823200
1	3.27281200	-1.86228800	1.65917200
6	0.34150000	1.99218600	-0.07069900
6	-0.60371900	2.55820300	-1.13142900
1	-0.43932800	2.07509900	-2.10131000
1	-0.39889400	3.62863100	-1.24315100
1	-1.65580300	2.44941100	-0.85311200
6	0.13039900	2.58521700	1.32353200
1	1.36827800	2.19724100	-0.38385500
1	0.29201700	3.66864800	1.28078300
1	0.84495500	2.16012600	2.03756900
1	-0.88479300	2.39579400	1.68263700
6	-2.19782700	-0.02634700	0.07409400
8	-2.94924900	-0.77660200	-0.72122400
8	-2.58723400	0.81556700	0.85940000
6	-4.40797000	-0.64773700	-0.59650700
6	-4.92543700	-1.49652100	0.54875600
1	-4.77508400	-0.99764800	-1.56265900
1	-4.65251000	0.41019200	-0.47508300
1	-4.65146900	-2.54817700	0.40945000
1	-6.01950600	-1.42499200	0.57584400
1	-4.53437800	-1.15039600	1.51106100

Table S46: total electronic energy, thermal correction to Gibbs free energy and atomic coordinates of conformer 5 of **7**

Total electronic energy (Hartree): -820.587508513

Thermal correction to Gibbs Free Energy (Hartree): 0.256086

Atomic coordinates:

8	1.04445500	-1.57041400	-0.22928400
7	-0.37965500	-1.61700000	-0.18711800
6	-0.74266000	-0.37709700	-0.04171100
7	0.30301000	0.50082700	0.02411400
6	1.46454200	-0.26135700	-0.10445300

7	2.66114600	0.15837900	-0.10633000
6	3.76213100	-0.81377200	-0.22207900
6	4.96280200	-0.11147800	-0.85938300
1	5.80139800	-0.80939100	-0.97332100
1	5.29374100	0.72754300	-0.23314800
1	4.70534100	0.28178900	-1.85049300
6	4.11621900	-1.38158100	1.16005100
1	3.46443600	-1.64971400	-0.87290500
1	4.41906700	-0.57335100	1.83831100
1	4.94670400	-2.09422700	1.08037900
1	3.26065200	-1.90293800	1.60573000
6	0.34990700	1.96224200	0.29723200
6	0.09054300	2.76987300	-0.97598700
1	0.77662100	2.46515900	-1.77446800
1	0.25832800	3.83286200	-0.76707600
1	-0.93787300	2.63839800	-1.32305200
6	-0.55262300	2.35049500	1.46929500
1	1.38831900	2.11661500	0.60089200
1	-0.34198600	3.39107100	1.73953600
1	-0.35127700	1.72215000	2.34432900
1	-1.61497600	2.28188000	1.21839700
6	-2.20094500	-0.01375200	-0.08443800
8	-2.92630800	-0.88962900	0.59877600
8	-2.61557200	0.94732700	-0.70218200
6	-4.38807600	-0.74405600	0.54855800
6	-4.94837900	-1.38696900	-0.70549700
1	-4.63213000	0.31875800	0.61723300
1	-4.72315300	-1.25350700	1.45342400
1	-4.58868800	-0.88242400	-1.60821900
1	-6.04237400	-1.31292000	-0.68229500
1	-4.67501900	-2.44675600	-0.75591200

Table S47: total electronic energy, thermal correction to Gibbs free energy and atomic coordinates of conformer 6 of **7**

Total electronic energy (Hartree): -820.588815315

Thermal correction to Gibbs Free Energy (Hartree): 0.255625

Atomic coordinates:

8	0.78465400	-1.57141500	-0.41023700
7	-0.63487200	-1.49127000	-0.44833000
6	-0.89531700	-0.23578700	-0.23275900
7	0.21729800	0.53902700	-0.05538800
6	1.31515200	-0.31334200	-0.17442900
7	2.54472800	-0.01818600	-0.08905400
6	3.54799800	-1.08806300	-0.22469400
6	4.83997300	-0.47094900	-0.76453100
1	5.61184700	-1.24024500	-0.89027100
1	5.22046400	0.29063400	-0.07110200
1	4.66886200	0.00691500	-1.73697700
6	3.77927000	-1.77029700	1.13148200
1	3.20258900	-1.84968200	-0.93992900
1	4.12522500	-1.03826400	1.87266400
1	4.54025200	-2.55563600	1.04107800
1	2.85682200	-2.22991500	1.50594300
6	0.28725200	1.97593100	0.32944200
6	0.92415200	2.13474500	1.71220600
1	0.38994100	1.53419400	2.45766500
1	0.86128900	3.18764800	2.00964300
1	1.97637400	1.83768300	1.70678600
6	0.98794300	2.78994500	-0.76007100
1	-0.75179200	2.29710400	0.38721700
1	0.93747100	3.85283900	-0.49787600
1	0.49393100	2.64958700	-1.72829700
1	2.03976900	2.50534700	-0.85509800
6	-2.30539400	0.27804700	-0.30702400
8	-3.17250300	-0.64234300	0.09209000
8	-2.57883300	1.39070100	-0.71747200
6	-4.59582600	-0.30207100	-0.02166500
6	-5.38159400	-1.49959300	0.46319300
1	-4.80172600	-0.06548800	-1.06973800
1	-4.77820300	0.58882100	0.58634000
1	-5.17099200	-2.38361100	-0.14891000
1	-6.45203300	-1.27463600	0.38897200

1	-5.14836400	-1.72722900	1.50933300

Table S48: total electronic energy, thermal correction to Gibbs free energy and atomic coordinates of conformer 7 of **7**

Total electronic energy (Hartree): -820.588101923

Thermal correction to Gibbs Free Energy (Hartree): 0.255967

Atomic coordinates:

8	0.96290800	-1.62694200	-0.37958000
7	-0.45477400	-1.70836100	-0.46848200
6	-0.86041000	-0.48268300	-0.31581600
7	0.15182200	0.41828400	-0.13330100
6	1.34097900	-0.30961200	-0.17691800
7	2.52608100	0.12405100	-0.05843100
6	3.64491200	-0.83282800	-0.11064400
6	4.88925600	-0.09286700	-0.60597400
1	5.74511800	-0.77619900	-0.66858100
1	5.14836200	0.72418200	0.08028000
1	4.71890100	0.33713200	-1.60065100
6	3.87822300	-1.45084500	1.27573900
1	3.42088400	-1.64575600	-0.81774900
1	4.10338300	-0.66652000	2.00993700
1	4.72382000	-2.14950600	1.24709400
1	2.99310800	-1.99978500	1.61858700
6	0.04606200	1.86784400	0.19078900
6	0.60564800	2.15282200	1.58676700
1	0.11574300	1.52289100	2.33836200
1	0.40978600	3.20175100	1.83659200
1	1.68462300	1.98144700	1.63022300
6	0.69438000	2.71250300	-0.90780900
1	-1.02367700	2.07197000	0.19785300
1	0.51769000	3.77241400	-0.69235800
1	0.25619000	2.48138200	-1.88541100
1	1.77390700	2.54309300	-0.95487200
6	-2.31479300	-0.13033400	-0.45537700
8	-3.08666300	-1.11863500	-0.02255400

8	-2.68805700	0.92110700	-0.94105100
6	-4.54105600	-0.95036800	-0.14440900
6	-5.09247600	-0.18636300	1.04399800
1	-4.91251400	-1.97596200	-0.17732600
1	-4.75542200	-0.45410900	-1.09385600
1	-4.84873600	-0.69678800	1.98242600
1	-6.18418000	-0.13189300	0.95412900
1	-4.69874300	0.83458200	1.08021200

Table S49: total electronic energy, thermal correction to Gibbs free energy and atomic coordinates of conformer 8 of **7**

Total electronic energy (Hartree): -820.588140942

Thermal correction to Gibbs Free Energy (Hartree): 0.256241

Atomic coordinates:

8	0.81487600	-1.62029400	-0.16585000
7	-0.60525400	-1.59892900	-0.08480800
6	-0.90602900	-0.33774600	0.01145700
7	0.17912800	0.49397600	-0.00019300
6	1.30266300	-0.32428300	-0.11931300
7	2.51959100	0.02523900	-0.17590200
6	3.55810700	-1.01388400	-0.28265000
6	4.76844600	-0.41269500	-1.00049600
1	5.56315600	-1.16093300	-1.10989800
1	5.16894500	0.43678900	-0.43163300
1	4.49240200	-0.05522400	-2.00014800
6	3.93637500	-1.52903800	1.11368100
1	3.19042700	-1.86414900	-0.87666100
1	4.30654900	-0.70481300	1.73701800
1	4.72528800	-2.28827700	1.04169200
1	3.07293600	-1.98040600	1.61685900
6	0.21148900	1.96680600	0.21792800
6	0.94518200	2.30493900	1.51807100
1	0.50091100	1.77009100	2.36555700
1	0.85352900	3.38101500	1.70439700
1	2.00740400	2.05275600	1.45796500
6	0.78664300	2.67940300	-1.00768600

1	-0.83396400	2.25283300	0.32503600
1	0.70844700	3.76226300	-0.85822300
1	0.22509500	2.41454000	-1.91083500
1	1.83961400	2.42488900	-1.15849200
6	-2.34013700	0.11084100	0.00324100
8	-3.12040100	-0.78037200	0.60114500
8	-2.69573100	1.15141000	-0.51766600
6	-4.57041900	-0.54653700	0.56420200
6	-5.16019600	-1.03444900	-0.74514000
1	-4.75419300	0.51713600	0.73337300
1	-4.93973500	-1.11944200	1.41627800
1	-4.76840700	-0.46775000	-1.59616500
1	-6.24835400	-0.90088900	-0.71439300
1	-4.94700800	-2.09850700	-0.89620200

The total electronic energies, thermal corrections to Gibbs free energy and atomic coordinates of **11** (Table S49-S54)

Table S50: total electronic energy, thermal correction to Gibbs free energy and atomic coordinates of conformer 1 of **11**

Total electronic energy (Hartree): -820.568008429

Thermal correction to Gibbs Free Energy (Hartree): 0.255691

Atomic coordinates:

7	1.11134900	-1.50243800	0.04366300
6	1.71928700	-0.36852000	0.01330100
8	-0.29985200	-1.44100400	0.12775900
6	1.05070100	0.97797600	0.06135600
8	1.70593400	2.00997800	0.11169200
7	-0.32088000	0.93405900	0.03847200
6	-1.02632100	-0.28108800	-0.02656300
7	-2.28413600	-0.33047600	-0.17256400
6	-2.98807700	-1.62046200	-0.22081100
6	-4.27097500	-1.43407700	-1.03392700
6	-3.29369400	-2.10421700	1.20420500
1	-2.36487400	-2.37715500	-0.71800500
1	-4.81919800	-2.38138000	-1.10653700
1	-4.92466700	-0.69121200	-0.55844300
1	-4.04362100	-1.09008600	-2.05038600
1	-2.37158000	-2.26055600	1.77563300
1	-3.90940800	-1.36640800	1.73447600
1	-3.84324400	-3.05315100	1.17337000
6	-1.11105200	2.21274100	0.03683400
6	-0.84052800	3.02891800	-1.22866900
6	-0.89695300	2.99156400	1.33663900
1	-2.14423000	1.87320800	0.00863700
1	-0.99695200	2.41751200	-2.12515400
1	-1.54594600	3.86753500	-1.26101500
1	0.17546200	3.43168100	-1.24724900
1	-1.08478800	2.35199900	2.20715900
1	0.11381700	3.40060000	1.40892200
1	-1.61088100	3.82316000	1.36524000
8	3.04308500	-0.30398300	-0.05930600

6	3.78922900	-1.55905300	-0.09547300
6	5.25738500	-1.20139100	-0.16429400
1	3.46183900	-2.12890600	-0.97206700
1	3.54568100	-2.13072500	0.80680400
1	5.84805400	-2.12453100	-0.19400900
1	5.47936600	-0.62010200	-1.06627300
1	5.56348000	-0.62377100	0.71515600

Table S51: total electronic energy, thermal correction to Gibbs free energy and atomic coordinates of conformer 2 of **11**

Total electronic energy (Hartree): -820.567860512

Thermal correction to Gibbs Free Energy (Hartree): 0.256125

Atomic coordinates:

7	-1.32269800	-1.34644800	-0.18070100
6	-1.86139200	-0.18330000	-0.06557900
8	0.08584900	-1.35592900	-0.32168900
6	-1.11862000	1.12362000	-0.09718800
8	-1.71897700	2.19080100	-0.11875800
7	0.25209200	1.00010300	-0.10771800
6	0.87265500	-0.25806200	-0.02899200
7	2.10463500	-0.39415700	0.21918400
6	2.72928600	-1.72364000	0.25608000
6	3.91392900	-1.66546700	1.22340400
6	3.17866600	-2.12588300	-1.15613000
1	2.01447700	-2.47474100	0.62010500
1	4.40090200	-2.64598300	1.29143600
1	4.65603500	-0.93275700	0.88060600
1	3.58469100	-1.37506400	2.22862300
1	2.32460500	-2.18782600	-1.84048700
1	3.88984500	-1.39186800	-1.55583700
1	3.67150500	-3.10566000	-1.13247800
6	1.06678100	2.26166900	-0.11035900
6	2.06897200	2.29278800	-1.26568500
6	1.69214400	2.53462800	1.26034600
1	0.31956500	3.03227800	-0.29789000
1	1.57600300	2.05394000	-2.21553500

1	2.47427500	3.30876300	-1.33951400
1	2.89776500	1.59845800	-1.11219400
1	0.93248500	2.48916300	2.04973300
1	2.48606600	1.82066200	1.49172400
1	2.11865400	3.54474700	1.25612400
8	-3.17492100	-0.04754300	0.07518900
6	-3.98959600	-1.25949500	0.08550600
6	-5.42900200	-0.82630000	0.25491600
1	-3.65051300	-1.89563600	0.91068100
1	-3.82713000	-1.79161400	-0.85830200
1	-6.06987700	-1.71574500	0.26701700
1	-5.56928300	-0.28664200	1.19824000
1	-5.74680600	-0.18292900	-0.57325900

Table S52: total electronic energy, thermal correction to Gibbs free energy and atomic coordinates of conformer 3 of **11**

Total electronic energy (Hartree): -820.566270873

Thermal correction to Gibbs Free Energy (Hartree): 0.256415

Atomic coordinates:

7	-1.47130400	-1.19456000	-0.10868800
6	-1.92999200	0.00361400	-0.20344500
8	-0.08196100	-1.30093700	0.14048600
6	-1.11709100	1.25698600	-0.02587200
8	-1.65335400	2.35702300	0.01900900
7	0.23852600	1.04661500	0.08645500
6	0.78841700	-0.24104900	-0.02759700
7	2.02540600	-0.44128000	-0.19546000
6	2.56827700	-1.80509000	-0.26669300
6	2.86557100	-2.32152900	1.14866000
6	3.83456500	-1.76896800	-1.12535500
1	1.84272000	-2.48285500	-0.73779800
1	3.29471900	-3.33001900	1.10186100
1	3.58451800	-1.66396200	1.65368300
1	1.95244300	-2.36499000	1.75349100
1	3.61326000	-1.39834900	-2.13374700
1	4.58735200	-1.10896600	-0.67502900

1	4.26441900	-2.77408700	-1.21564200
6	1.12160300	2.25088500	0.24348900
6	1.87103900	2.57631900	-1.05135400
6	2.02675500	2.13883500	1.47153000
1	0.40766200	3.05288400	0.42918900
1	1.17668200	2.63678400	-1.89778300
1	2.35707300	3.55280200	-0.93929900
1	2.63593000	1.82859800	-1.27375400
1	1.44533000	1.86776400	2.36073700
1	2.82330000	1.40490600	1.33198200
1	2.48381200	3.11916200	1.65032400
8	-3.21242000	0.23798100	-0.46061900
6	-4.12427800	-0.89659200	-0.61085600
6	-4.54293600	-1.46344200	0.73401200
1	-3.65034400	-1.64864900	-1.24937200
1	-4.97390200	-0.46182700	-1.14076600
1	-5.28604000	-2.25282800	0.56689100
1	-3.69605900	-1.89847000	1.27333600
1	-5.00097700	-0.68567000	1.35542700

Table S53: total electronic energy, thermal correction to Gibbs free energy and atomic coordinates of conformer 4 of **11**

Total electronic energy (Hartree): -820.566196673

Thermal correction to Gibbs Free Energy (Hartree): 0.256049

Atomic coordinates:

7	-1.47671000	-1.15036500	-0.44021000
6	-1.94798900	0.03347000	-0.26370700
8	-0.06560800	-1.24234500	-0.51879800
6	-1.12159700	1.28607000	-0.15379400
8	-1.64955600	2.39051700	-0.12017700
7	0.23706300	1.07122800	-0.10503000
6	0.76960900	-0.22891000	-0.09005200
7	1.97111300	-0.46923200	0.22154800
6	2.50212400	-1.83906400	0.19052900
6	3.62584500	-1.93698500	1.22494300
6	3.00962100	-2.16884700	-1.22077800

1	1.71695400	-2.56123200	0.45445400
1	4.04004000	-2.95249100	1.24461300
1	4.43576300	-1.23679800	0.98235300
1	3.25536700	-1.69773400	2.22927400
1	2.19709900	-2.11918200	-1.95489100
1	3.79231300	-1.46113400	-1.52214700
1	3.43229600	-3.18087600	-1.24467100
6	1.12912400	2.27175300	0.02846800
6	2.20102100	2.31168900	-1.06216600
6	1.68318400	2.40904700	1.44909100
1	0.44606700	3.10238200	-0.14732700
1	1.75341300	2.17179400	-2.05330200
1	2.67463300	3.30017100	-1.03993900
1	2.97268200	1.55369500	-0.91215400
1	0.87305900	2.37077000	2.18696500
1	2.40714800	1.62340500	1.67766800
1	2.18061400	3.38198100	1.53968500
8	-3.25568500	0.25590200	-0.18651500
6	-4.17564500	-0.87792300	-0.28655800
6	-4.27568500	-1.63763000	1.02426000
1	-3.85898200	-1.51828300	-1.11552500
1	-5.12452300	-0.40345000	-0.54413900
1	-5.03670600	-2.42064900	0.91931700
1	-3.32772500	-2.11430300	1.29168400
1	-4.58066000	-0.96810400	1.83643200

Table S54: total electronic energy, thermal correction to Gibbs free energy and atomic coordinates of conformer 5 of **11**

Total electronic energy (Hartree): -820.566401912

Thermal correction to Gibbs Free Energy (Hartree): 0.255852

Atomic coordinates:

7	1.26818700	-1.35987300	-0.20296300
6	1.80591900	-0.19167700	-0.23388800
8	-0.13165100	-1.38834200	0.00277300
6	1.06791200	1.10681100	-0.04869500

8	1.66509800	2.17275300	0.01589200
7	-0.29637800	0.98044100	0.03358000
6	-0.93336100	-0.27028300	-0.05665600
7	-2.19264400	-0.38903100	-0.13428300
6	-2.82148600	-1.71584900	-0.21180000
6	-4.15335100	-1.57055700	-0.95135400
6	-3.02419300	-2.28305200	1.20063900
1	-2.18148100	-2.40921500	-0.77548500
1	-4.64684700	-2.54559900	-1.04601300
1	-4.82509100	-0.89375100	-0.40734000
1	-3.99954600	-1.16436500	-1.95860400
1	-2.06612400	-2.40955000	1.71774200
1	-3.65376100	-1.60997500	1.79666900
1	-3.51800700	-3.26127400	1.14929100
6	-1.15584100	2.20592100	0.16850700
6	-1.03477400	3.10555300	-1.06310800
6	-0.88290100	2.92379400	1.49230900
1	-2.16701200	1.80564900	0.19933900
1	-1.22920600	2.53626700	-1.97966800
1	-1.78589000	3.90083100	-0.99042400
1	-0.04750200	3.56895700	-1.13654200
1	-0.96222700	2.22664300	2.33484400
1	0.10502400	3.39052400	1.51064200
1	-1.63929100	3.70615800	1.62604300
8	3.10898300	-0.03417500	-0.43712500
6	3.95075800	-1.22050700	-0.59920500
6	4.28055100	-1.86231300	0.73662400
1	4.84578500	-0.82330700	-1.08168600
1	3.45373000	-1.91571500	-1.28284100
1	4.97831800	-2.69119900	0.56533600
1	4.76242000	-1.13932200	1.40435400
1	3.38772300	-2.26143800	1.22743800

Table S55: total electronic energy, thermal correction to Gibbs free energy and atomic coordinates of conformer 6 of **11**

Total electronic energy (Hartree): -820.566363847

Thermal correction to Gibbs Free Energy (Hartree): 0.256382

Atomic coordinates:

7	-1.26723900	-1.35988400	-0.29213500
6	-1.81795200	-0.19870000	-0.23884800
8	0.14748700	-1.37321100	-0.27935000
6	-1.07730300	1.10826900	-0.14924900
8	-1.67194200	2.17729400	-0.17100300
7	0.28539300	0.98662200	-0.03731000
6	0.92292100	-0.26656600	-0.01612300
7	2.16674800	-0.39423900	0.19174200
6	2.79811900	-1.72214300	0.19370000
6	4.04255900	-1.65802700	1.08194700
6	3.15567100	-2.13482900	-1.24159400
1	2.10985800	-2.47176400	0.60908600
1	4.53487800	-2.63741400	1.12246200
1	4.75998500	-0.92588700	0.68882800
1	3.77835300	-1.36379900	2.10510200
1	2.25977200	-2.20209000	-1.86939400
1	3.83944300	-1.40297800	-1.69050000
1	3.64997000	-3.11416600	-1.24308200
6	1.13751500	2.21688000	0.10313500
6	0.81763800	2.96785500	1.39725700
6	1.05909800	3.08437400	-1.15517100
1	2.14723300	1.81951300	0.18120700
1	0.87303400	2.29403400	2.26034800
1	1.56355800	3.75944300	1.53497800
1	-0.17333500	3.42816000	1.37032800
1	1.27892400	2.49016700	-2.05001100
1	0.07783300	3.55103100	-1.27192300
1	1.81335800	3.87646200	-1.07935100
8	-3.13815500	-0.05604000	-0.26778000
6	-3.98075700	-1.25033800	-0.34631400
6	-4.13282500	-1.92053100	1.00754900
1	-3.56667400	-1.92606600	-1.10097500
1	-4.93390100	-0.85635600	-0.70404100
1	-4.83774500	-2.75519000	0.90907400

1	-3.18124300	-2.31658700	1.37493000
1	-4.53483100	-1.21580700	1.74417600
